# Mechanochromic Break Points Control the Toughness of Entangled Polyphenylenes

**DOI:** 10.1021/acsmacrolett.4c00810

**Published:** 2025-02-10

**Authors:** Annina Missikewitsch, Hartmut Komber, Till Biskup, Michael Sommer

**Affiliations:** † Institute for Chemistry, Polymer Chemistry, Chemnitz University of Technology, Straße der Nationen 62, 09111 Chemnitz, Germany; ‡ Leibniz-Institut für Polymerforschung Dresden e.V., Hohe Straße 6, 01069 Dresden, Germany; § Institut für Chemie, Physikalische Chemie, Universität Rostock, Albert-Einstein-Straße 27, 18059 Rostock, Germany

## Abstract

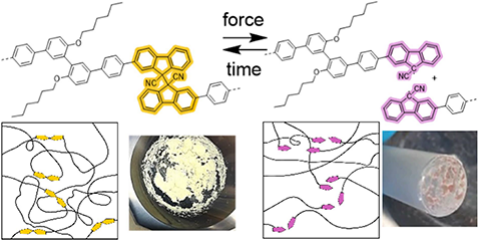

Toughness engineering of a kinked polyphenylene (P*mmp*P) is demonstrated by using mechanochromic molecular break points. Varying amounts of thermally stable yet mechanically labile difluorenylsuccinonitrile (DFSN) motifs incorporated into P*mmp*P allow to largely tune mechanical failure of the specimen. While strain at break values of pristine P*mmp*P reach up to 300%, an increasing concentration of DFSN break points leads to a strongly decreasing and predictable strain at break. Homolytic bond scission of DFSN and formation of colored DFSN radicals is characterized by *in situ* UV–vis spectroscopy, which allows us to discern regions of necking and strain hardening during tensile testing. The formation and lifetime of radicals is further probed by EPR spectroscopy, suggesting reversibility of bond scission and thus the possibility to design tough materials with predicted failure and self-healing properties.

Molecular break points using covalently installed mechanophores in polymers allow to direct mechanical deformation and stress to weak points of the chain, giving rise to mechanoresponsive behavior and eventually generating new functions.^1−4^ Potential applications span a wide range of possibilities, including optical detection and quantification of forces to map stress distributions of a macroscopic specimen,^5^ access to unusual reaction pathways or products,^6−9^ self-healing and reinforcement of gels,^10^ or the release of small molecules in biomedical applications.^11^ While mechanochromic polymers display changes in stress by color change, which may not necessarily be accompanied by chain scission,^12−14^ mechanical activation of molecular break points by chain cleavage may^7−9,15−19^ or may not^20^ produce colored products.^21^ For example, spiropyran-based force probes indicate stress by bond cleavage, but the resulting merocyanine can isomerize back to SP and ensure that the molar mass of the chain remains unchanged. If, however, distinct applications require mechanical deformation to produce both color change and a change in mechanical properties (i.e., viscosity, strength, toughness, strain at break), colored mechanophores that function via covalent bond scission need to be used.

Colored, stabilized mechanoradicals open up pathways to force responsiveness by both chain cleavage and visible color generation. While the reduction in molar mass upon chain scission changes mechanical properties, the resulting radicals can be detected optically or by means of electron paramagnetic resonance (EPR) spectroscopy.^4^ Modulating the reactivity of mechano-generated radicals can furthermore be a useful handle to control subsequent reactions related to cross-linking processes,^22^ hydrogen abstractions,^22,23^ and antioxidant properties.^24,25^ Along these lines, diarylbenzofuranones (DABBF)^26^ and difluorenylsuccinonitrile (DFSN)^27^ exhibit a variable temperature range for their activation. DFSN has been used as a functional cross-linker^22,28,29^ to toughen^28^ or self-strengthen^22,30^ materials or mapping stress^30,31^ of networks. A further useful property is the low reactivity of DFSN radicals toward oxygen,^27^ which opens up possibilities for recombination and thus self-healing under ambient conditions. DFSN further exhibits a reasonably high thermal stability up to 130 °C, which is ideal regarding its synthesis and incorporation into polymers at elevated temperature.^27,32,33^ However, DFSN motifs carry hydroxymethyl groups,^22,27−29,34^ which limit the range of polymer chemistries and matrices to be used for covalent incorporation and thus the range of smart materials accessible.

To make use of the unique properties of the DFSN motif also in more rigid and tough polymers, we here report the synthesis of a brominated DFSN derivative, 2,2′-dibromo-9,9′-bi-9*H*-fluorene-9,9′-dicarbonitrile (DFSN-Br_2_), and its incorporation into the tough and glassy poly(*meta–meta–para*)phenylene, P*mmp*P, via Suzuki polycondensation (Scheme 1).^35^ P*mmp*P with hexyloxy side chains is amorphous with a glass transition temperature *T*_g_ of ∼110 °C, a Young’s modulus of 0.9 GPa^35^ and strain at break values up to 300%,^35^ provided that molar mass is sufficiently high. These properties arise from the high share of *meta* linkages in the backbone,^35,36^ which cause an entanglement molar mass of *M*_e_ = 4.8 kg·mol^–1^.^37,38^ The large strain at break values of P*mmp*P render this polymer ideal to investigate stress-related phenomena *in situ* during tensile testing.^14,39,40^ With increasing concentration of DFSN in the P*mmp*P chain, the strain at break values continuously decrease. The unique beneficial toughness of P*mmp*P is entirely eliminated for DFSN concentrations as low as ∼5%. *In situ* UV–vis absorption spectroscopy during tensile testing indicates that the first fraction of DFSN is activated within the necking region. A second, possibly larger, fraction of DFSN mechanophores fails within the strain hardening regime, where the force per chain increases. EPR spectroscopy of ground powders indicates a half-life of the radicals of 0.94 d at room temperature ascribed to recombination.

**Scheme 1:**
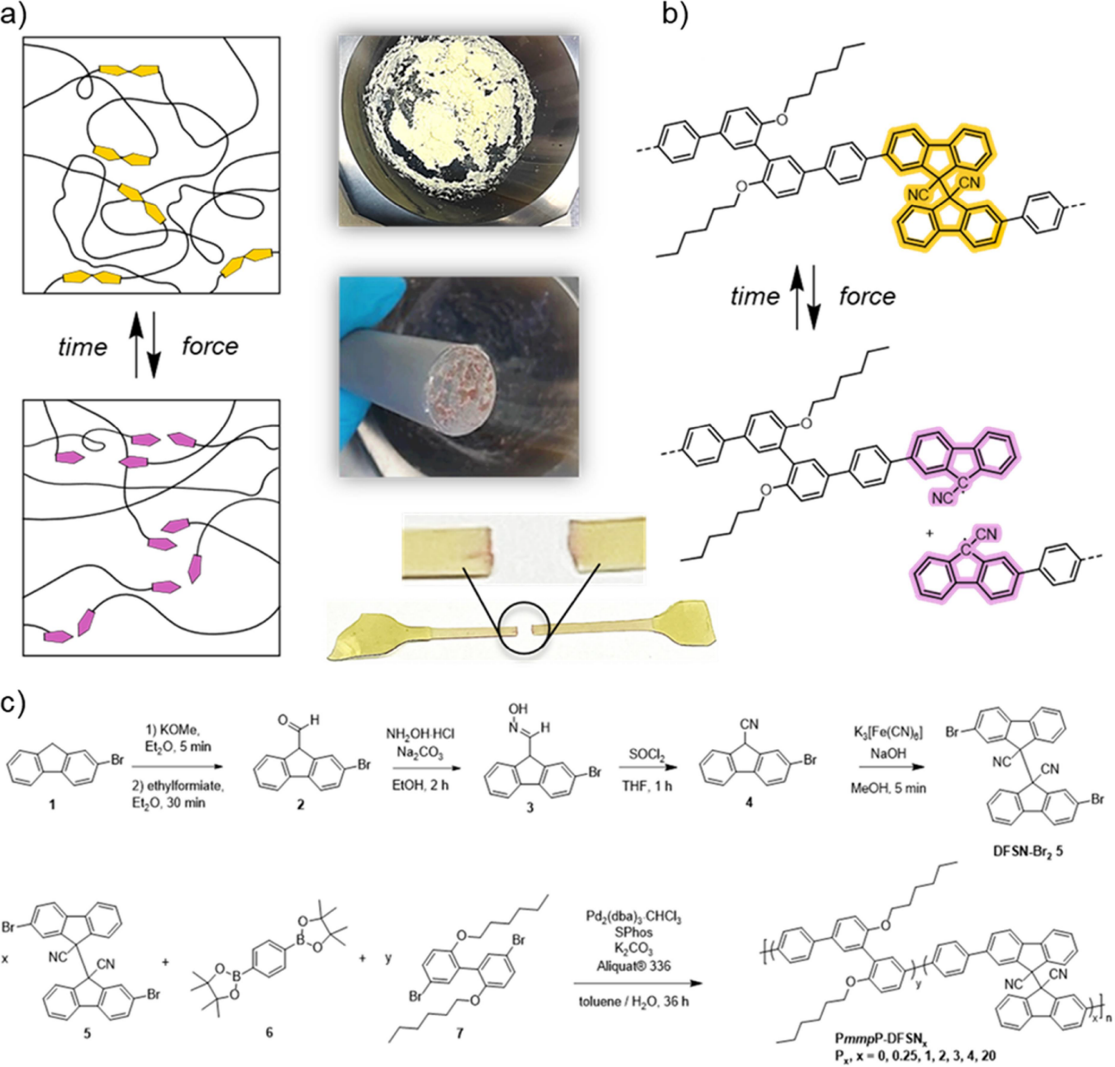
Overview of the Synthesis and Mechanical Properties of Tough Polyphenylenes P*mmp*P-DFSN_*x*_ with Varying mol % *x* of Mechanochromic Break Points Based on Difluorenylsuccinonitrile (DFSN) and mol % *y* of Biphenyl Comonomer **7** such that *x* + *y* = 100 mol % (a) Schematic representation of an entangled P*mmp*P-DFSN_*x*_ network before and after mechanical activation. The two photographs show the color of the material before and after grinding; (b) Chemical structures of P*mmp*P-DFSN_*x*_ before and after homolytic bond cleavage with photographs of a failed stress–strain specimen; (c) Synthesis and copolymerization of DFSN-Br_2_**5** via Suzuki polycondensation resulting in P*mmp*P-DFSN_*x*_ with varying concentrations of DFSN (given by *x* = mol % of the initial mixture of dihalides).

We synthesized tough P*mmp*P with varying amounts of DFSN units incorporated statistically into the backbone, furnishing a series of P*mmp*P-DFSN_*x*_, with *x* and *y* being the molar fractions of DFSN-Br_2_ and biphenyl monomer **7**, respectively, such that *x* + *y* = 100 mol % in the monomer feed (Scheme 1, Table 1). The yellow color of as-prepared P*mmp*P-DFSN_*x*_ changes upon mechanical activation via tensile testing or grinding, resulting in a pink color (Scheme 1a,b).

**Table 1:** Molecular Parameters and Elongation at Break Values of the Different P*mmp*P-DFSN_*x*_ Samples

sample P_*x*_	feed ratio DFSN-Br_2_/Biph-Br_2_	DP_*n*_^a^/avg DFSN units per chain^b^	*M*_n,SEC_^c^ [kg·mol^–1^]	*M*_w,SEC_^c^ [kg·mol^–1^]	yield [%]	ε_B(max)_ [%]
P_0_	0/100	160/0.0	68	113	92	277
P_0_^d^	0/100	110/0.0	48	79	64	220
P_0.25_	0.25/99.75	160/0.5	67	114	85	226
P_1_	1/99	130/1.5	56	94	98	148
P_2_	2/98	230/4.5	99	175	93	160
P_3_	3/97	180/5.5	79	148	97	122
P_4_	4/96	180/7.0	78	148	96	36
P_20_	20/80	20/4.0	10	19	72	

a Calculated from number-average molecular weights determined via size exclusion chromatography *M*_n,SEC_.

b Estimated from monomer feed ratio and *M*_n,SEC_ and rounded to 0.5.

c Determined via SEC at 40 °C in tetrahydrofuran and using polystyrene calibration.

d Reference sample with lower *M*_n_ and *M*_w_ due to short polymerization time of 12 h.

The DFSN-Br_2_ monomer **5** was prepared in five steps starting from 2-bromofluorene **1** using a modified procedure from Sakai et al.^27^ First, **1** was deprotonated with potassium methoxide and quenched with ethyl formate to generate the aldehyde functionality in 9-position (Scheme 1c). The aldehyde **2** was further converted into oxime **3**, which was directly used to obtain the corresponding nitrile **4** via oxidation with SOCl_2_. The final dimerization step to furnish DFSN-Br_2_**5** with potassium ferrocyanide(III) as oxidant was optimized by using degassed solvents to achieve a relatively high yield of 36% (see Supporting Information for details).

DFSN-Br_2_**5** is a mixture of two stereoisomers due to the chiral 9,9′ carbons. Furthermore, steric interactions result in strongly preferred *gauche* conformations for the central C9–C9′ bond.^41,42^ This produces rotamers that interconvert by rotation around this bond. A study on the rotational isomerism for the unsubstituted DFSN was reported by Lam et al.^42^ The rate of the inversion process influences the appearance of the NMR signals and results in temperature-dependent NMR spectra. A detailed characterization of DFSN-Br_2_**5** via NMR spectroscopy including information on stereoisomers is presented in the Supporting Information.

DFSN-Br_2_**5** was copolymerized with 1,4-benzenediboronic acid bis(pinacol)ester **6** and 5,5′-dibromo-2,2′-bis(hexyloxy)-1,1′-biphenyl **7**(14,35) to get P*mmp*P-DFSN_*x*_ with 0.25 mol % < *x* < 20 mol % (Scheme 1c, Table 1). Statistical incorporation of DFSN is expected due to an anticipated similar reactivity of **5** and **7**.

The detection and quantification of DFSN units in P*mmp*P-DFSN_*x*_ was challenging due to the low intensity of the relevant signals, but also due to the dynamic processes mentioned above causing very broad ^1^H NMR signals at room temperature. The sample with the highest DFSN content, P*mmp*P-DFSN_20_, shows few broad DFSN signals at 30 °C and was measured in different solvents and at different temperatures to improve DFSN detection (Figure 1). At elevated temperatures, the signals from the DFSN unit shift and are likely overlapped by backbone signals and thus cannot be clearly identified. Slowing down the dynamic processes at −30 °C leads to well-detectable signals of protons 7 and 8, whereas other signals are overlapped. The assignment is based on the ^1^H NMR study of the DFSN-Br_2_ monomer (see Supporting Information). Under similar conditions, semiquantification of the covalently incorporated DFSN was possible also for the polymers with low content, confirming similarity between monomer feed ratios and copolymer composition (see Supporting Information, Figure S13, and Table 1). The additional DFSN units are not expected to significantly change the physical properties of the P*mmp*P chain in the concentration range used. For example, the glass transition temperatures of pristine P*mmp*P and P*mmp*P-DFSN_4_ are 121 and 118 °C (see Figure S15), confirming this assumption.

**Figure 1:**
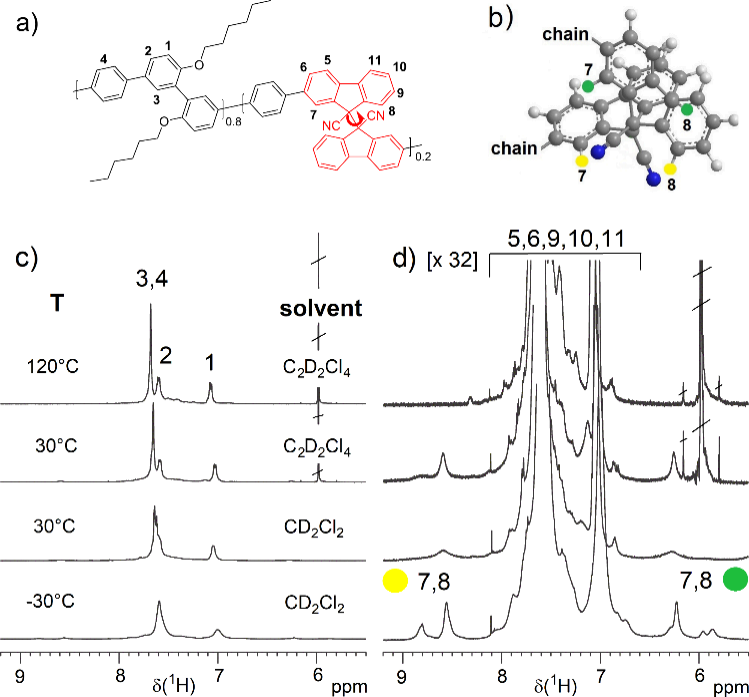
^1^H NMR spectroscopic characterization of P*mmp*P-DFSN_20_ having the largest fraction of DFSN. (a) Chemical structure of P*mmp*P-DFSN_20_ with atom numbering, (b) 3D structure of a *meso*-DFSN unit with marked protons **7** and **8** next to the CN groups (yellow) and near the shielding region of aromatic rings (green). (c) Aromatic region of the ^1^H NMR spectrum as a function of temperature and (d) same region as shown in (c) but magnified by a factor of 32 in order to assign the signals to protons **7** and **8** in the two different environments (−30 °C spectrum, slow exchange). For further information from NMR spectroscopy, see the Supporting Information.

The series of P*mmp*P-DFSN_*x*_ with varying DFSN content allows investigation of their mechanical properties as a function of the density of break points. Specimens for tensile testing were prepared by punching dog bone-shaped specimens from solution-cast films. For pristine P*mmp*P with a high molar mass (*M*_w_ > 90 kg/mol), large strain at break values up to ε_B_ ∼ 300% are reported.^35^ This is the case of P_0_, which delivered ε_B_ = 277%, while all other samples P_*x*_ with *x* = 0.25–4 showed significantly smaller values of ε_B_ (Figure 2a,b, Table 1). As strain at break also depends on molar mass, an effort was made to maintain a similar level of *M*_w_ values, as far as this was possible for products made by polycondensation (note that *M*_n_ values are strongly influenced by the shape of the SEC curve of these kinked polymers and are therefore less meaningful). Figure 2b depicts the combined effects of the DFSN content and molar mass variation on ε_B_. This explains why the two samples with *M*_w_ < 100 kg/mol, P_0_ and P_1_, fail at relatively small values of ε_B_ compared to the other samples. However, an overall clear trend of an increasing DFSN content causing strongly decreasing strain at break from ε_B_ = 277% (P_0_) to ε_B_ = 36% (P_4_) emerged. P_20_ did not show film-forming properties and could therefore not be tested. Stress at break values were less dependent on DFSN content (Figures 2a and S17). P_3_ and P_4_ showed a faint pink color upon failing, which was only visible at the breaking edge of the sample, while intensity of color formation was generally weak along the necked areas, being hardly visible by the naked eye (Scheme 1b, Figure 2c).

**Figure 2:**
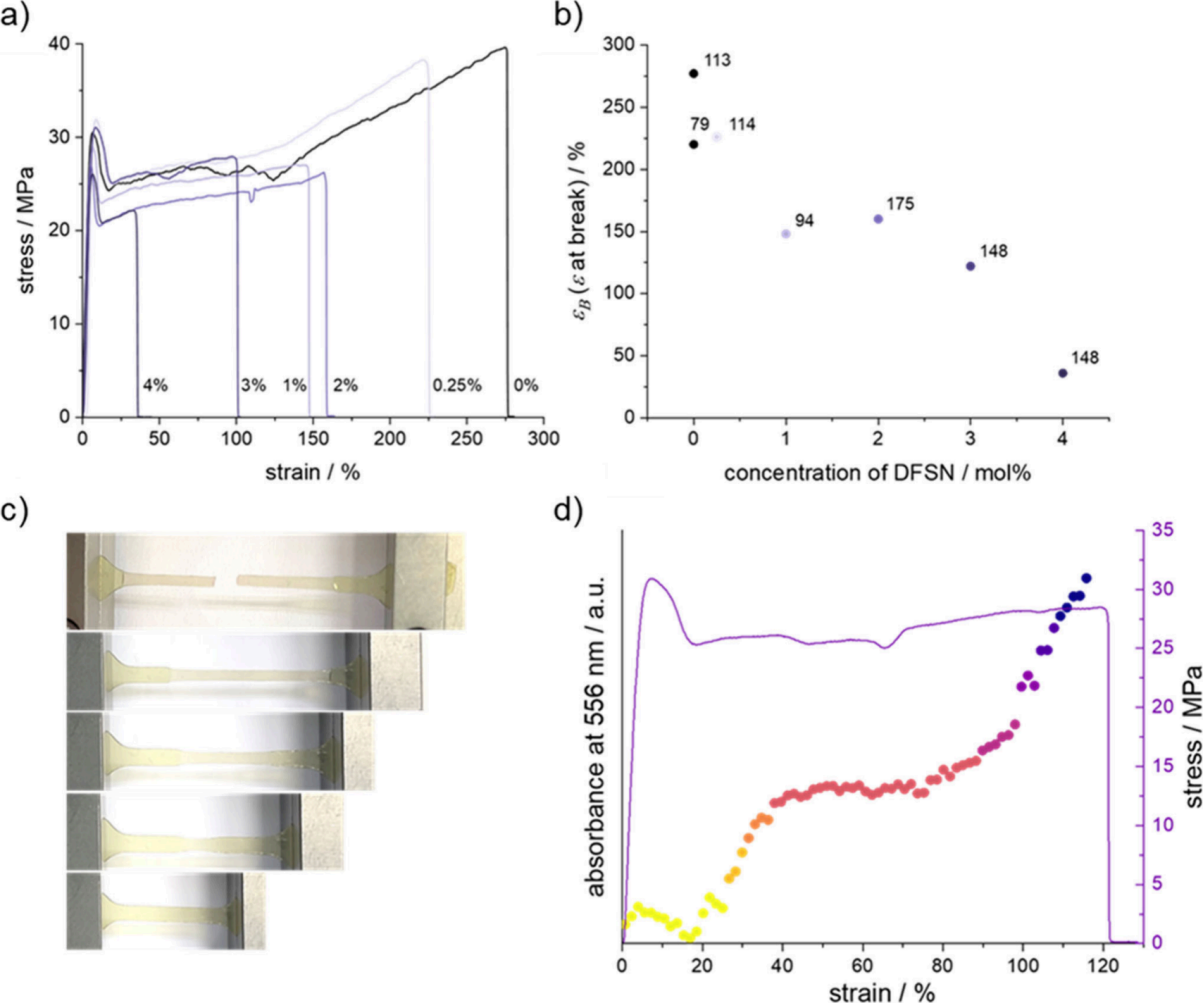
Opto-mechanical characterization of P*mmp*P-DFSN_*x*_. (a) Representative samples of stress–strain behavior with varying concentrations of DFSN_*x*_, *x* = 0, 0.25, 1, 2, 3, and 4 mol %. The specimen thickness was between 110–150 μm. (b) Representative strain at break versus concentration of DFSN in P*mmp*P-DFSN_*x*_. The numbers indicate the weight-average molecular weights, *M*_w_, which additionally influence the correlation. (c) Photographs of bone shaped specimens under load during the stress–strain experiment, indicating that detection by the naked eye is barely possible. (d) *In situ* absorbance at 556 nm as a function of strain of P*mmp*P-DFSN_3_ showing that DFSN is a suitable force probe and that stress transduction occurs after sample necking.

To further monitor DFSN cleavage, the mechano-optical characterization of P*mmp*P-DFSN_*x*_ (*x* = 2, 3) was carried out via tensile testing with *in situ* UV–vis absorption in transmission. Pristine P*mmp*P does not show a significant absorbance beyond 500 nm. Although the overall intensity of the pink DFSN radical was weak, absorbance at 556 nm^27^ clearly showed mechanically induced formation of DFSN radicals. However, scattering effects caused by thick specimens complicated analysis (see Supporting Information). P*mmp*P-DFSN_3_ developed an absorbance peak with increasing strain (Figures S17–S19) that vanished following specimen failure and relaxation (Figure S21). This suggests recombination of DFSN radical-terminated chains, which is remarkable in light of the high *T*_g_ and large molar mass compared to room temperature and the entanglement molecular weight of P*mmp*P,^38^ respectively. Following the absorbance of P*mmp*P-DFSN_3_ at 556 nm as an example, DFSN cleavage can be correlated with different regions of the stress–strain curve (Figure 2d). The onset of absorption at 556 nm occurs as soon as the specimen enters the necking region where plastic flow allows the transduction of macroscopic force to the chain. During the first half of the necking region, the absorbance at 556 nm remained mostly constant.

At the end of the necking and in the beginning of the strain hardening region, absorbance further increased with saturation of this process finally not being reached due to early failure of the specimen. This second increase in absorbance is explained by increasing stress in the entangled network.

To gain information about the formation and also stability of DFSN radicals over time and thus further insight into DFSN mechanochemistry, P*mmp*P-DFSN_20_ was ground and investigated via EPR spectroscopy. EPR spectra were taken from bulk powders before and after grinding, as well as after different times after the grinding process. Figure 3a shows the absence of an EPR signal before grinding as well as an intense signal with hyperfine splitting directly after grinding. The splitting and experimental Landé *g*-factor of 2.003 align with the DFSN derivative as described by Sakai et al.^27,43^ The intensity of the EPR spectra over time decreased continuously until the signal vanished after ∼5 days (Figure 3b), along with the discoloration of the ground powder. Fitting the decay with a monoexponential fit yields a half-life of 0.94 days for this sample, i.e., for the ground bulk powder of low molar mass sample P_20_. This decrease may be explained by the kinetics of a first order reaction.

**Figure 3:**
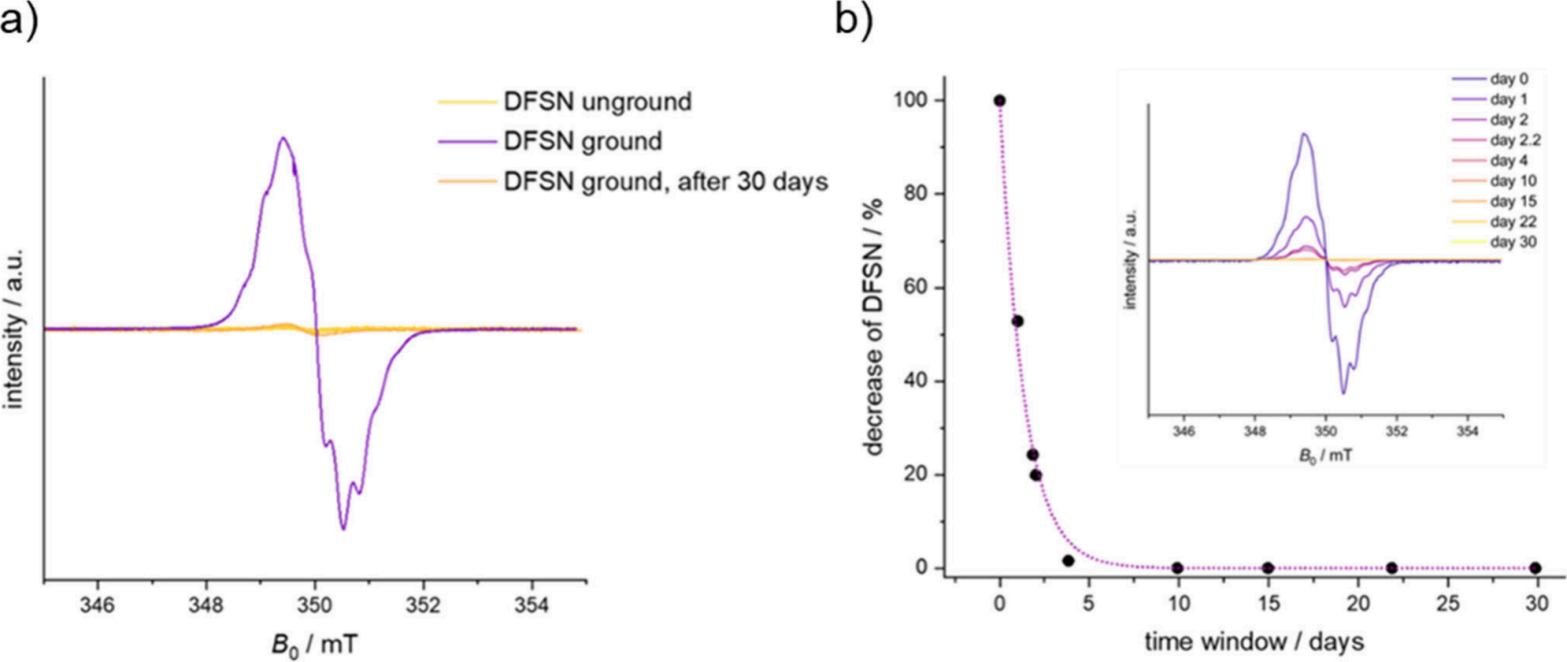
(a) Electron paramagnetic resonance (EPR) spectra of P*mmp*P-DFSN_20_ bulk powder before and at different times after the grinding process. The magnetic field was calibrated by measuring TEMPOL.^44^ The intensity was normalized for every measurement with Cu(acac)_2_ measured under the same conditions. For detailed measurement parameters, see the Supporting Information. (b) Decrease of the intensity of the EPR signal over a time period of 30 days. The decrease was estimated with a function of *y* = 100 × exp(−*x*/*t*), with *t* = 1.35.

Since DFSN radicals exhibit remarkable stability toward oxygen and as the shape of the decaying EPR spectra does not suggest the formation of a new species with unpaired electrons, we assume the decaying intensity of the EPR spectra to result from recombination of radicals. Such process was suggested on the basis of *in situ* UV–vis spectroscopy following specimen failure (Figure S21), but can be clearer seen and quantified by EPR spectroscopy.

However, reactions that produce noncolored and EPR-silent products other than those from DFSN radical recombination cannot be entirely ruled out. While spectroscopic characterization of such reaction via NMR spectroscopy is challenging due to the issues of spectroscopic detection mentioned (*vide supra*), we have furthermore subjected ground powders of P*mmp*P_20_ to SEC analysis, where a broadening of the molecular weight distribution toward both longer as well as shorter chains, was observed (see Figure S25). Upon addition of tetrahydrofuran, the pink color instantaneously disappeared, possibly due to an increased chain mobility facilitating recombination. The appearance of chains longer than those present in the original SEC distribution can only be explained by cleavage of DFSN furnishing scrambled lengths of recombined chains. Upon recombination of longer segments, larger molar masses than those present in the original molecular weight distribution may form, suggesting chain segment recombination at the expense of DFSN radicals. This is in line with literature data that supports a robust DFSN radical capable of selective C–C bond breaking and recombination without major side reactions.^26,34^

In conclusion, we have designed tough polyphenylenes P*mmp*P-DFSN_*x*_ with varying amounts of mechanochromic molecular break points based on the difluorenylsuccinonitrile (DFSN). A brominated DFSN monomer was successfully prepared and incorporated into a tough polyphenylene via Suzuki polycondensation. In a series of P*mmp*P-DFSN_*x*_ copolymers, the composition according to the monomer feed was confirmed by NMR spectroscopy. The mechanical properties are a strong function of DFSN content, whereby 4 mol % DFSN in the copolymer almost entirely eliminated the unique toughness behavior of P*mmp*P. More specifically, strain at break can be controlled through DFSN density in the chain, allowing one to predict material failure. The investigation of mechano-optical behavior showed that two fractions of covalently incorporated DFSN were activated in the necking and strain hardening regime of the stress strain region. While the pink color of the DFSN radicals is rather weak rendering detection of low concentrations challenging, the DFSN radicals are remarkably stable and EPR-active, which opens up providing opportunities for analytical detection. Yet, while DFSN radicals are robust, they also recombine and thus open up possibilities for the design of tough materials with controlled mechanical and potentially self-healing properties.

## Supporting Information Available

Experimental procedures, NMR and IR spectra, MS data, thermal properties, EPR spectra, UV–vis absorbance spectra and information for tensile testing, and SEC curves (PDF)

## Abbreviations

DABBF: diarylbibenzofuranones
DFSN: difluorenylsuccinonitrile
EPR: electron paramagnetic resonance
P*mmp*P: poly(*meta*-*meta*-*para*)phenylene
PS: polystyrene
SEC: size exclusion chromatography
TEMPOL: 4-hydroxy-2,2,6,6-tetramethylpiperidin-1-oxyl.
