# Supplementary material for: Mechanochromic Break Points Control the Toughness of Entangled Polyphenylenes
Source: ACS Macro Lett. 2025 Feb 10;14(3):243–9. doi: 10.1021/acsmacrolett.4c00810 (PMC11924313; doi:10.1021/acsmacrolett.4c00810)
Supplement: Supplementary file 1 — mz4c00810_si_001.pdf [file mz4c00810_si_001.pdf]

Supporting Information

Mechanochromic break points control toughness of entangled polyphenylenes

Annina Missikewitsch[1], Hartmut Komber[2], Till Biskup[3] and Michael Sommer[1,*]

[1]Institute for Chemistry, Polymer Chemistry, Chemnitz University of Technology, Straße der Nationen 62, 09111 Chemnitz, Germany

[2] Leibniz-Institut für Polymerforschung Dresden e. V., Hohe Straße 6, 01069 Dresden, Germany

[3] Institut für Chemie, Physikalische Chemie, Albert-Einstein-Straße 27, Universität Rostock, 18059 Rostock, Germany

Contact (email): michael.sommer@chemie.tu-chemnitz.de

Table of contents

1. General information 3

2. Synthesis 6

  2-Bromo-9*H*-fluorene-9-carboxaldehyde oxime (3) 6

  2-Bromo-9*H*-fluorene-9-carbonitrile (4) 7

  [2,2’-Dibromo-9,9’-bi-9*H*-fluorene]-9,9’-dicarbonitrile (DFSN-Br_2 5) 8

  P*mmp*P-DFSN$_x$, x= 0.25, 1, 2, 3, 4, 20 mol% 9

3. NMR data, spectra and discussion 11

  3.1 NMR spectra of 2-bromo-9*H*-fluorene-9-carbonitrile (4) 11

  3.2 NMR spectra and data of isomers’ mixture of DFSN-Br_2 5 and discussion of the dynamic processes 12

4. SEC curves 21

5. Thermal characterization 22

6. Mechano-optical characterization 23

7. EPR data and reversibility 27

8. Supporting references 31

1. General information

Chemicals. All chemicals, reagents and solvents were purchased from commercial sources and used as received unless otherwise noted. Diethyl ether, methanol, tetrahydrofuran, toluene, and water were degassed by purging with argon for minimum 1 hour. Phenylene and biphenylene monomers were prepared according to Kempe *et al*.[1]

NMR Spectroscopy. NMR spectra were recorded on a Bruker AVANCE III 500 spectrometer (^{1}H: 500.1 MHz, ^{13}C: 125.8 MHz) at different temperatures (see data and spectra). DMSO-d_6, CD_2Cl_2 and $C_2D_2Cl_4$ were used as solvents. The spectra were referenced to the solvent peak (DMSO-d_6: $\delta(^1H)$ = 2.50 ppm, $\delta(^{13}C)$ = 39.6 ppm; CD_2Cl_2: $\delta(^1H)$ = 5.33 ppm, $\delta(^{13}C)$ = 53.7 ppm; $C_2D_2Cl_4$: $\delta(^1H)$ = 5.98 ppm). 2D NMR spectra were recorded to support signal assignment.

Size Exclusion Chromatography (SEC). Molecular weights were measured on a Shimadzu system comprising a 5 µm precolumn and three SDplus columns with pore sizes ranging from 10^2 to 10^4 Å (Polymer Standards), connected in series with a RID20A RI detector and a SPD20AV photodiode array UV-vis detector (Shimadzu). Calibration was done with polystyrene standards having molar masses between 682 and 552000 g·mol^{-1}. THF was used as eluent at 40 °C with a flow rate of 1.0 mL·min^{-1}.

UV-vis Spectroscopy. Thin-film UV-vis absorption spectra were measured on a Flame-S UV-vis spectrometer from Ocean Optics with an integration time of 4 ms and 250 scans to average, controlled by the OceanView 1.5.2 software. All UV-vis spectra were measured in transmission. Thin-film absorption spectra were smoothed via Savitzky-Golay filter considering 21 pts and normalized. Baseline correction for thin film spectra was performed on basis of asymmetric least squares smoothing with an asymmetric factor of 10^{-5}, a threshold of 0.007 and a smoothing factor of 7^{20}.

IR-Spectroscopy. Infrared (IR) analysis was performed on an Alpha II FT-IR spectrometer from Bruker.

Matrix Assisted Laser Desorption Ionization (MALDI) TOF. The MALDI-TOF spectra were taken using a BRUKER autoflex maX MALDI-TOF instrument in reflectron positive mode. The laser of this instrument is a smartbeam II with a wavelength of 355 nm. The software for measuring and evaluating the spectra is flexControl 3.4 and flexAnalysis 3.4. N2200 (naphthalene bithiophene copolymer, M_n = 23 kg·mol^{-1}) was used as matrix.[2] Samples were

prepared on a standard sample plate (Bruker "MTP 384 target plate ground steel BC" Part-No.: 8280784).

Differential Scanning Calorimetry (DSC). DSC measurements were performed on a DSC 2500 (TA Instruments) in aluminum standard pans under nitrogen atmosphere with a heating-/cooling rate of 10 K·min^{-1}. Sample masses were between 3.5 and 5.5 mg.

Preparation of films. For each film 200 mg of polymer sample were dissolved in 2 mL dichloromethane (DCM) and stirred for 15 min. The solution was filtered and cast into a petri dish (ø = 35 mm). The sample was covered with an inverted beaker and the solvent was evaporated overnight in a fume hood at room temperature. The dumbbell shaped specimens for mechanical testing were die-cut from the film using a standard punching tool (ISO 37 type 4) having a width of 2 mm. Typical film thicknesses were between 110-150 µm.

Tensile Testing (uniaxial). Stress-strain experiments were carried out on a Linkam TST-350. A standard specimen shape according to DIN 53504 type 3 equal to ISO 37 type 4 was used with film thicknesses between 110 and 150 µm. The stress shown is the engineering stress considering the constant cross-section area of the specimen's mid-point at 0% strain. The true stress before breaking is therefore expected to be significantly higher due to continuous thinning of the specimen during stretching. Strain shown is the engineering strain representing the percentage elongation in relation to the initial specimen gauge length of 15 mm. The displacement or strain rate was 83.33 mm·min^{-1}.

Electron Paramagnetic Resonance (EPR) Spectroscopy. EPR spectroscopy was carried out using a Bruker EMX-10/12 spectrometer with a X-band microwave bridge (type ER041XG) and rectangular shaped cavity (type ER4119HS). The spectrometer was controlled using WinEPR version 3.03. Wilmad EPR quartz tubes (4 mm outer and 3 mm inner diameter) were used. All measurements were performed at room temperature. EPR spectra were field-corrected by measuring TEMPOL (solution in toluene, about 30 µM) with known Landé-factor g = 2.0057.[3] For the semi-quantitative analysis, $Cu(acac)_2$ (solid powder homogeneously mixed with SiO_2 powder for diamagnetic dilution in about 1:20 ratio) was used for intensity calibration. All measured samples (exept TEMPOL) were used as pure solids, to exclude effects due to altered characteristics. All spectra were frequency-corrected to 9.81 GHz microwave frequency. For the semi-quantitative analysis, double integration of the EPR signal was performed using Origin (Version Origin Pro 2023b) and its maximum determined, which was divided by the maximum of the double integral of the $Cu(acac)_2$ sample. The microwave power

was 1.6 mW for the DFSN and $Cu(acac)_2$ samples and 0.6 mW for the TEMPOL reference. The modulation frequency was 100 kHz for all samples. The modulation amplitude was 0.1 mT for all samples.

2. Synthesis

2-Bromo-9*H*-fluorene-9-carboxaldehyde oxime (3)

2-Bromofluorene **1** (25.71 g, 104.89 mmol, 1.00eq.) was dissolved in diethyl ether (510 mL, 0.20 M, distilled, degassed) under Ar. To the mixture, KOMe (10.97 g, 156.49 mmol, 1.49 eq.) was added and the mixture was stirred for 5 min. After that, ethylformiate (25.35 mL, 23.32 g, 314.82 mmol, 3.00 eq.) was added quickly and stirred for 30 min at room temperature. The mixture was added to water (600 mL) and washed with diethyl ether (5 × 70 mL). The aqueous phase was acidified with con. HCl (3 mL) and extracted with diethyl ether (2 × 100 mL). The combined organic phases were washed with water (2 × 100 mL) and dried over $NaSO_4$. The crude product **2** was filtrated and after removal of the solvent under reduced pressure an orange solid was obtained (29.13 g). The crude **2** product was directly used without further purification (13.85 g, 50.70 mmol, 1.00 eq.) and dissolved in ethanol (400 mL, 0.13 M, abs.) under Ar. To the mixture, hydroxylamine hydrochloride (8.86 g, 127.50 mmol, 2.51 eq.) was added and the mixture was stirred for 2 h at room temperature. The crude product **3** was filtrated and the removal of the solvent under reduced pressure led to a yellow solid (4.19 g, yield 30% mass %) containing two isomers *(E)* and *(Z)* in a molar ratio of 60:40 as determined from the ^{1}H NMR spectrum of the crude product **3** in DMSO-d_6.

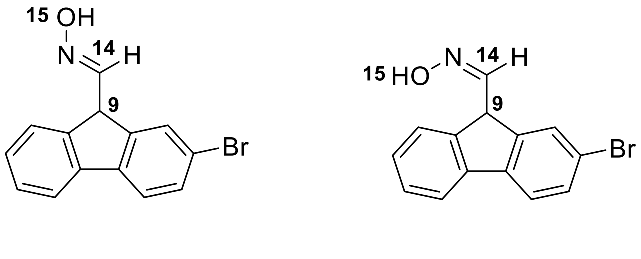

E-aldoxime **Z**-aldoxime

^{1}H NMR (500 MHz, DMSO-d_6): characteristic signals of the substance - δ 11.57 (s, 1H; 15_Z), 10.93 (s, 1H; 15_E), 7.34 (d, 6.8 Hz, 1H; 14_E), 6.60 (d, 6.2 Hz, 1H; 14_Z), 5.61 (d, 6.2 Hz, 1H; 9_Z), 4.84 ppm (d, 6.8 Hz, 1H; 9_E).

^{13}C NMR (126 MHz, DMSO-d_6): characteristic signals of the substance - δ 147.4 (10_E), 146.8 (10_Z), 48.2 (9_E), 44.3 ppm (9_Z).

MALDI-ToF (*m/z*): 288.09 $[M]^+$.

2-Bromo-9*H*-fluorene-9-carbonitrile (4)

The raw product of 2-bromo-9*H*-fluorene-9-carboxaldehyde oxime **3** (4.05 g, 1.00 eq.) was dissolved in THF (81 mL, 0.17 M, dried over molecular sieves 4Å, degassed) under Ar. $SOCl_2$ (1.66 g, 13.98 mmol, 1.02 mL, 0.99 eq.) was added and the mixture was stirred 1 h at room temperature. The mixture was then added to water (100 mL) and the aqueous phase was extracted with ethyl acetate (3 × 30 mL). The organic phase was washed with brine (3 × 30 mL) and dried over $MgSO_4$. The solvent was removed under reduced pressure and the crude product was purified via optimized column chromatography (petrol ether / ethyl acetate, 10:1; + 5% NEt_3). A white solid was obtained (0.4952 g).

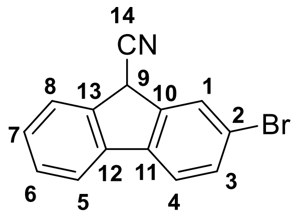

^{1}H NMR (500 MHz, CD_2Cl_2): δ 7.88 (s, 1H; 1), 7.79 (d, 7.8 Hz, 1H; 5), 7.73 (d, 7.6 Hz, 1H; 8), 7.69 (d, 7.9 Hz, 1H; 4), 7.65 (d, 8.2 Hz, 1H; 3) 7.53 (t, 7.5 Hz, 1H; 6);), 7.47 (t, 7.5 Hz, 1H; 7), 4.99 ppm (s, 1H; 9).

^{13}C NMR (126 MHz, CD_2Cl_2): δ 140.2 (11), 140.1 (10), 140.0 (12), 138.1 (13), 132.7 (3), 129.7 (6), 128.9 (7), 128.6 (1), 125.3 (8), 122.2 (4), 121.8 (2), 120.9 (5), 117.7 (14), 37.1 ppm (9).

IR: $\tilde{\nu}$ 2234 cm^{-1} (ν(C≡N)).

Elemental analysis / % found (calculated): N% 5.17 (5.19), C% 62.18 (62.25), H% 3.05 (2.99).

[2,2'-Dibromo-9,9'-bi-9*H*-fluorene]-9,9'-dicarbonitrile (DFSN-Br_2 5)

In a beaker, potassium hexacyanoferrate(III) (0.4811 g, 1.461 mmol, 1.10 eq.) was dissolved in aqueous NaOH (0.8000 g, 20.000 mmol, 4.00 mL, 5 M, 15.04 eq.). The optimized and purified 2-bromo-9-cyanofluorene **4** (0.3593 g, 1.330 mmol, 1.00 eq.) was dissolved in dry and degassed methanol (90 mL, 0.01 M) under Ar. After complete dissolution, the basic solution of potassiumhexacyanoferrate(III) was added and the stirring was continued for 5 min at room temperature. The solvent was filtered off and the product was washed with water (2 × 10 mL). As product, a white solid remained, consisting of the equimolar mixture of racem and meso form. (0.2600 g, 36% yield). This equimolar mixture was used for the polymer syntheses. The diastereomers could be separated in small quantities for NMR analysis due to their different solubility in CD_2Cl_2. The solubility of the *meso* diastereomer is lower than that of the *racemic* diastereomer.

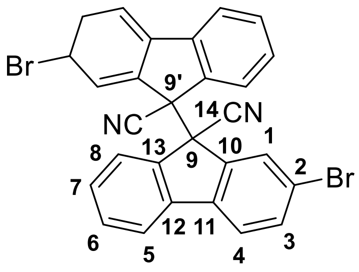

^{1}H and ^{13}C NMR data and spectra are reported and discussed in Chapter **3**.
IR: $\tilde{\nu}$ 2238 cm^{-1} (ν(C≡N)).

MALDI-ToF (*m/z*): 537.52 $[M]^{-}$.

Due to occurring internal C-C bond cleavage under MALDI-TOF-conditions an intense signal of 269.96 *m/z* is observed. This corresponds to the molecular peak of the radical form of **4.**

P*mmp*P-DFSN$_x$, x= 0.25, 1, 2, 3, 4, 20 mol%

The preparation of the monomers 1,4-benzenediboronic acid bis(pinacol)ester (Ph-BPin$_2$) and 5,5'-dibromo-2,2'-bis(hexyloxy)-1,1'-biphenyl (Biph-Br$_2$) as well as for the pristine poly-(*meta,meta,para*)-phenylene (P*mmp*P) P$_0$ was according to KEMPE *et al.*[1] and RAISCH *et al.*[4]

P$_{0.25}$ to P$_{20}$ (values for P$_3$ as chosen representative). A screw cap vial was loaded with Ph-BPin$_2$ (0.2155 g, 0.6521 mmol, 1.00 eq.) and Biph-Br$_2$ (0.3241 g, 0.6326 mmol, 0.97 eq.), DFSN-Br$_2$ (0.0105 g, 0.0196 mmol, 0.03eq.), Pd$_2$(dba)$_3$·CHCl$_3$ (0.0017 g, 0.0016 mmol, 0.0025 eq.), SPhos (0.0021 g, 0.0050 mmol, 0.0077eq.), K$_2$CO$_3$ (0.5747 g, 4.1582 mmol, 6.38 eq.) and 3 drops Aliquat 336 and the atmosphere was changed to argon (3 ×). Toluene (degassed, 1.73 mL) was added and the reaction mixture was stirred for 5 min at 70 °C. After the addition of H$_2$O (degassed, 2.08 mL, 0.31 mol·l^{-1}), the mixture was further stirred vigorously at 70 °C. After 24 h, the heat was shut off and the cooled reaction mixture was diluted with toluene and precipitated in methanol (80 mL). All polymers P$_{0.25}$ to P$_{20}$ were synthesized according to this procedure, whereby the ratio of Biph-Br$_2$ and DFSN-Br$_2$ was adjusted to the desired polymer composition. For sample P$_0$ the same procedure was used, without addition of DFSN-Br$_2$. Each precipitated sample was fractionated via Soxhlet extraction subsequently with methanol, diethyl ether and chloroform and freed from solvent under reduced pressure. The fractions studied in this work are listed in **Table S1**.

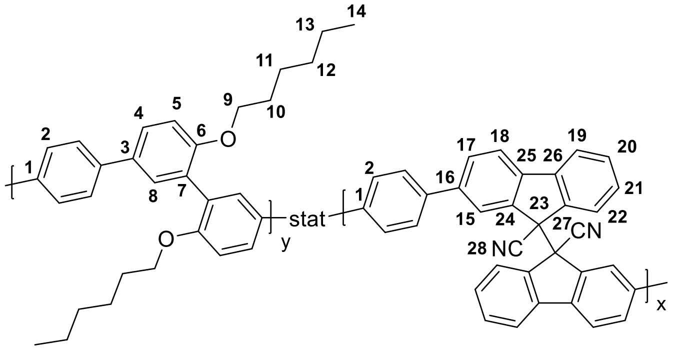

P*mmp*P-DFSN$_x$
P$_x$, x = 0, 0.25, 1, 2, 3, 4, 20 (molar content of DFSN)
x + y = 100 mol%

^{1}H NMR (500 MHz, CD$_2$Cl$_2$, 30°C): δ 8.60 (v br; 15/22), 8.2-7.1 (very broad signals of 17-21), 7.65 (2), 7.63 (8), 7.60 (4), 7.06 (5), 6.30 (v br; 15/22), 3.99 (9), 1.67 (10), 1.32 (11), 1.21 (12,13), 0.80 ppm (14). Note: The signals of the DFSN comonomer are very broad. For discussion of these effects see Chapter **3** Supporting Discussion.

^{13}C NMR (126 MHz, CD_2Cl_2, 30°C): δ 156.6 (6), 142-137 (16, 24-27), 139.2 (1), 132.7 (3), 130.4 (8), 130-120 (15,17-22), 128.7 (7), 127.1 (2), 127.0 (4), 118.9 (28), 112.7 (5), 68.9 (9), ~54 (23, overlapped by solvent signal), 31.9 (12), 29.6 (10), 26.0 (11), 22.9 (13), 14.1 ppm (14). Note: Most of the signals of the DFSN comonomer are broadened and could not be assigned by 2D NMR techniques. The reported signal positions were calculated on the basis of the signal assignments for DFSN-Br_2 and substituent chemical shift increments of Br and phenyl (C_{15} – C_{18}, C_{24}, C_{25} from C_1 – C_4, C_{10}, C_{11}) or taken from DFSN-Br_2 (C_{19} – C_{22}, C_{26} - C_{28} from C_5 – C_9, C_{12} – C_{14}).

Table S1. Molar ratio of DFSN-Br_2 and Biph-Br_2 comonomers and respective molar masses and yields of synthesized Polymers P*mmp*P (**P_0**) and P*mmp*P-DFSN$_x$ (**$P_{0.25\text{-}20}$**).

**Sample**	**equivalents**			**M_n (SEC)**	**M_w (SEC)**	**Yield**	**Dispersity**
**P_x**	**DFSN-Br_2**	**/**	**Biph-Br_2**	**[kg · mol^{-1}]**	**[kg · mol^{-1}]**	**mass %**	**M_w/M_n**
**P_0**	0.00	/	1.00	68	113	92	1.66
**P_0**	0.00	/	1.00	48	79	64	1.64
**$P_{0.25}$**	0.0025	/	0.99	67	114	85	1.69
**P_1**	0.01	/	0.99	56	94	98	1.68
**P_2**	0.02	/	0.98	99	175	93	1.77
**P_3**	0.03	/	0.97	79	148	97	1.87
**P_4**	0.04	/	0.96	78	148	96	1.91
**P_{20}**	0.2	/	0.8	10	19	72	1.88

3. NMR data, spectra and discussion

3.1 NMR spectra of 2-bromo-9*H*-fluorene-9-carbonitrile (4)

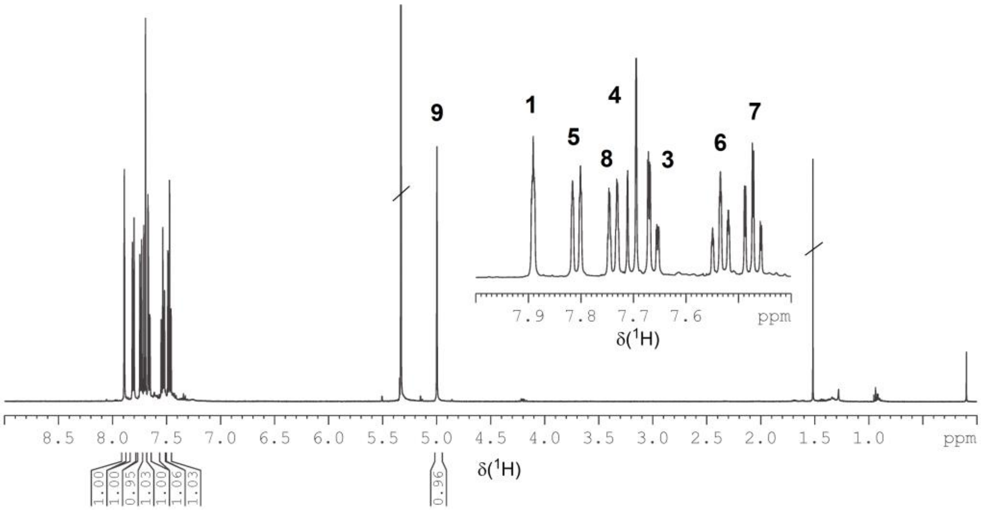

Figure S1. ^{1}H NMR spectrum of 2-bromo-9*H*-fluorene-9-carbonitrile **4** in CD_2Cl_2 at 30°C. The inset shows the enlarged signal region of aromatic protons.

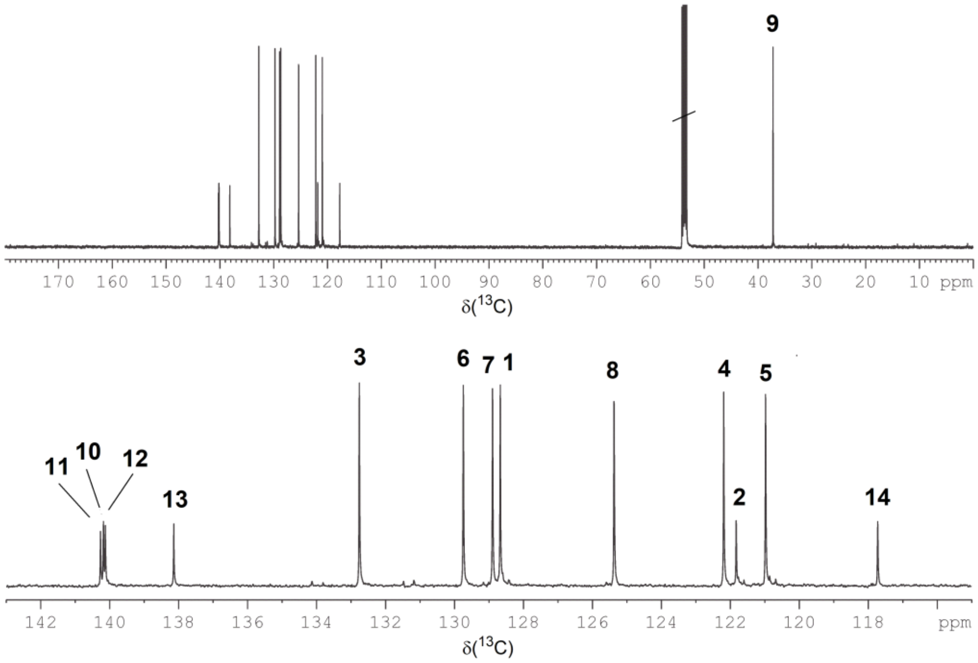

Figure S2. ^{13}C NMR spectrum of 2-bromo-9*H*-fluorene-9-carbonitrile **4** in CD_2Cl_2 at 30°C (overview and enlarged signal region of aromatic carbons).

3.2 NMR spectra and data of isomers' mixture of DFSN-Br_2 5 and discussion of the dynamic processes

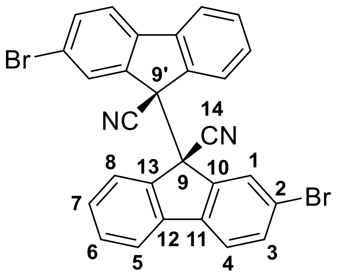

rac

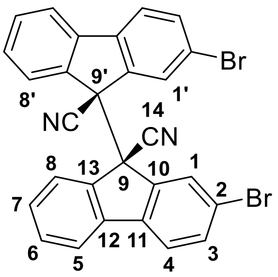

meso

[2,2'-dibromo-9,9'-bi-9*H*-fluorene]-9,9'-dicarbonitrile (DFSN-Br_2 5) seems to be a new compound according a Scifinder substance search. The compound contains two chiral centres: the carbons 9 and 9'. This allows the compound to form two diastereomers, each represented by two enantiomers: *rac* [(R,S)/(S,R)] and *meso* [(R,R)/(S,S)]. In an achiral environment, only the diastereomers can be distinguished by NMR spectroscopy.

Here we report the ^{1}H and ^{13}C NMR data of both diastereomers and some results of low-temperature measurements.

The conformational behaviour of 9,9'-bifluorenyl compounds was first studied in 1978 by DOUGHERTY *et al.* using NMR spectroscopy[5] and was further investigated in 1981 by OLAH *et al.*[6] using low-temperature NMR measurements. The main feature is the hindered rotation about the central C-C bond. It was found that the preferred conformation is characterized by the *gauche* arrangement of the 9,9' substituents.

NMR data of (R,S)/(S,R) isomer (meso)

^{1}H NMR (500 MHz, CD_2Cl_2, 30°C): δ 7.60 (d, 2H; 3), 7.52 (d, 2H; 5), 7.44 (t, 2H; 6), 7.42 (d, 2H; 4), ~7.4 (v br; 1), 7.26 (br t, 2H; 7), ~7.2 ppm (v br; 8).

^{1}H NMR (500 MHz, CD_2Cl_2, -60°C): δ 8.57 (s, 1H; 1‘), 8.45 (d, 1H; 8), 7.86 (d, 1H), 7.69 (2H), 7.63 (2H), 7.38 (d, 1H), 7.29 (d, 1H), 7.29 (d, 1H), 7.18 (t, 1H), 6.78 (t, 1H), 5.95 (s, 1H; 1), 5.76 ppm (d, 1H; 8‘). Note: Only signals from one rotamer are observed. Only verified signal assignments are given. Protons 1 and 8’ seem to experience a shielding by the ring current of the opposite aromatic ring, whereas 1’ and 8 seem to experience a deshielding by the nitrile group.

^{13}C NMR (126 MHz, CD_2Cl_2, 30°C): δ 140.4 and 140.3 (11, 12), 139.5 (10), 137.6 (13), 134.1 (3), 131.2 (6), 128.7 (7), 128.6 (1), 125.4 (8), 121.8 (4), 121.7 (2), 120.7 (5), 117.9 (14), 53.9 ppm (9).

Chem3D 17.0 (PerkinElmer Informatics Inc.) calculations at the MM2 level in combination with the dihedral driver module (rotation around the C-C bond) were performed to obtain a rough impression of the conformation of the observed rotamers of **DFSN-Br$_2$ 5**.

Figure S3 shows two rotamers for the *racemic* diastereomer with slightly different conformational energy. Both symmetrical rotamers are characterised by a *gauche*-like arrangement of the nitrile groups but differ, for example, in the aromatic hydrogen atoms next to the nitrile group. In the rotamer with lower energy (left; almost same energy for dihedral angles -75° and -130°) the protons 8 of both fluorene moieties are directed towards the nitrile groups, while in the rotamer with higher energy (right; almost same energy for dihedral angles 73° and 128°) protons 1 of both fluorene moieties are directed towards the nitrile groups. This is consistent with the ^{1}H NMR results at low temperature: Proton 8 of the major rotamer is deshielded, i.e., next to the nitrile group, while proton 1 for the minor isomer is deshielded.

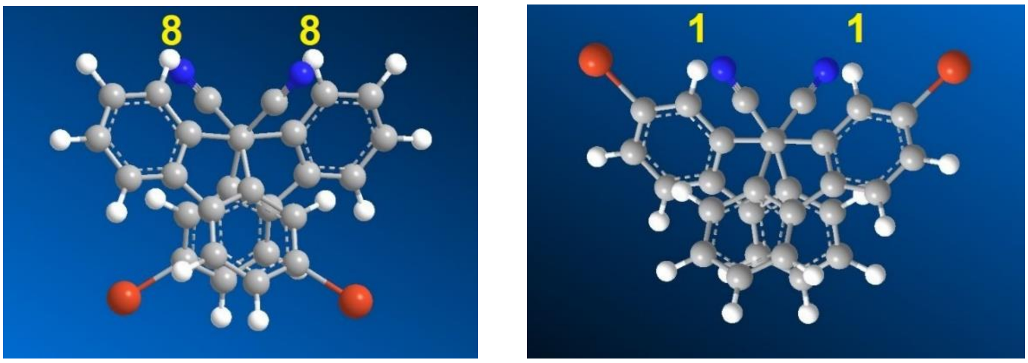

Figure S3. Structures of two rotamers of ***rac*-DFSN-Br$_2$ 5** from MM2 calculations (Chem3D 17.0).

The calculations for the *meso* diastereomer (**Figure S4**) yield only symmetrical rotamers that differ in the CN-C-C-CN dihedral angle (-121°, -61°, 82° and 139°) but have almost the same conformational energy. All these structures have the same characteristic feature: one nitrile group is directed to a proton 1 and the other to proton 8 of the other fluorene unit. This is consistent with the ^{1}H NMR results at low temperature: One rotamer is observed in which one proton 1 and one proton 8 are deshielded.

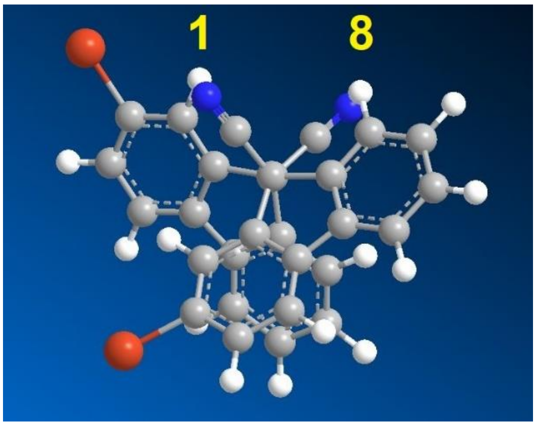

Figure S4. Structure of a rotamer of ***meso*-DFSN-Br$_2$ 5** from MM2 calculations (Chem3D 17.0).

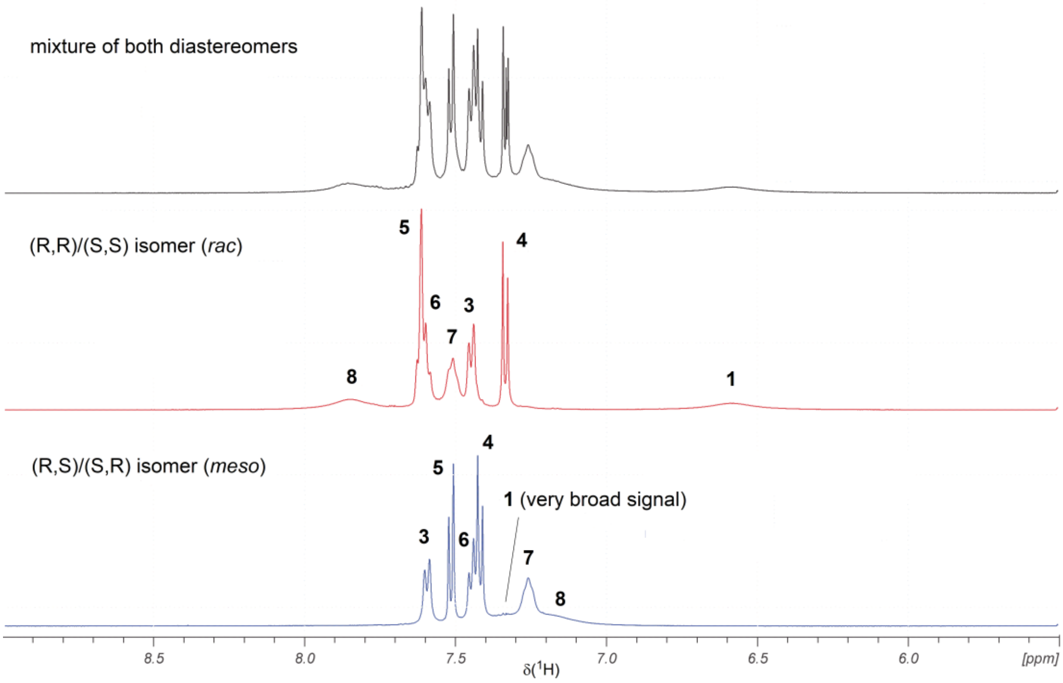

Figure S5. ^{1}H NMR spectrum of the synthesized mixture of diastereomers of **DFSN-Br$_2$ 5** and the isolated two diastereomers in CD_2Cl_2 at 30°C.

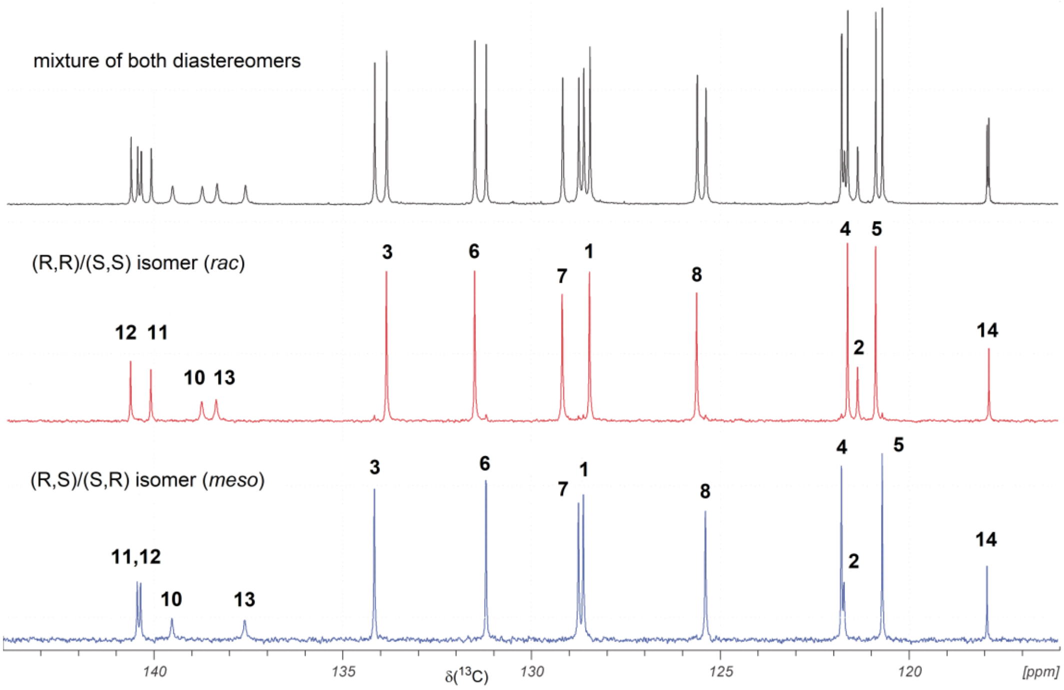

Figure S6. ^{13}C NMR spectrum of the synthesized diastereomer mixture of **DFSN-Br$_2$ 5** and the two isolated diastereomers in CD_2Cl_2 at 30°C. The signals of aliphatic carbon 9 appear at 54.1 ppm (*rac*) and 53.9 ppm (*meso*) in the region of the solvent signal.

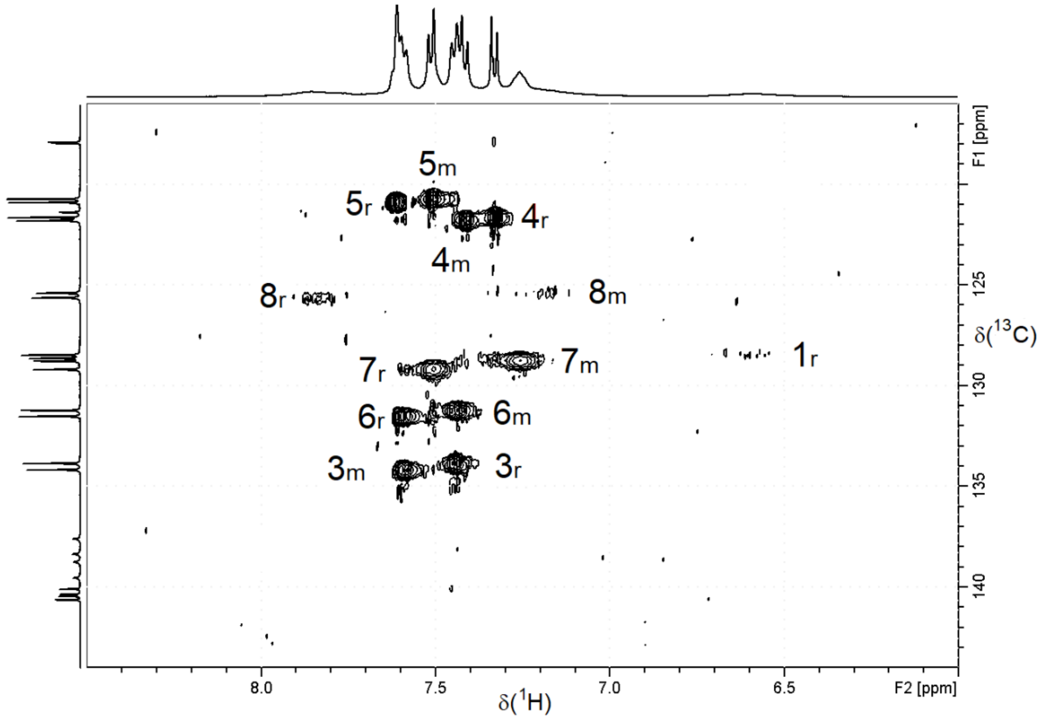

Figure S7. HSQC spectrum of the synthesized diastereomer mixture of **DFSN-Br$_2$ 5** in CD_2Cl_2 at 30°C.

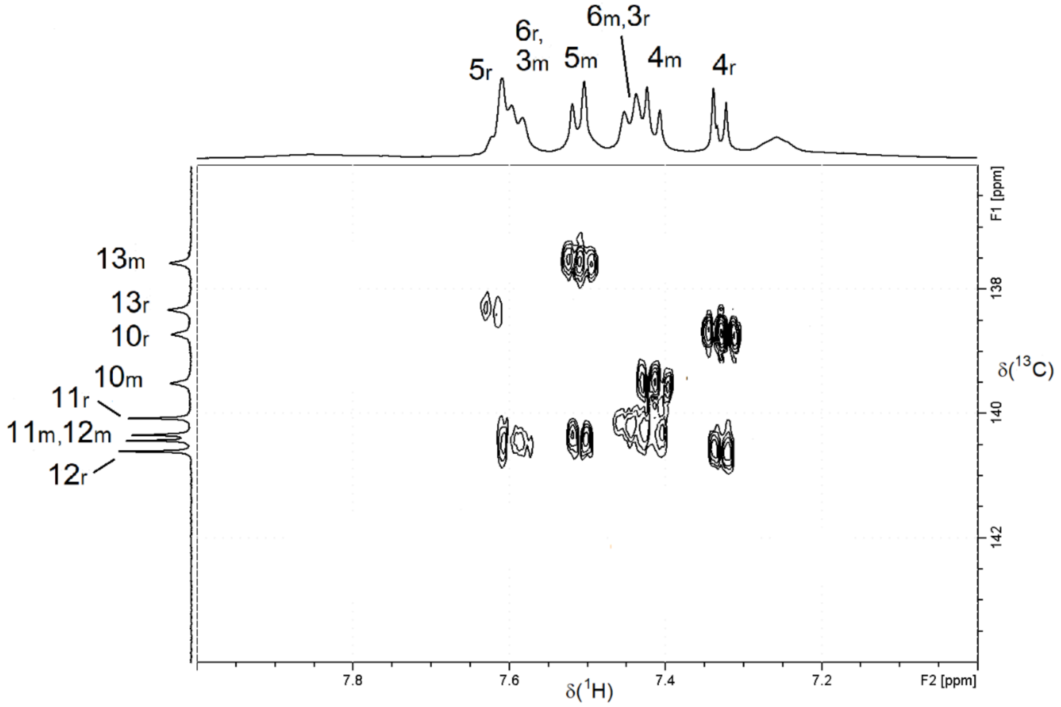

Figure S8: HMBC spectrum (region) of the synthesized diastereomer mixture of **DFSN-Br$_2$ 5** in CD_2Cl_2 at 30°C. The observed correlations make it possible to assign the signals of non-protonated carbons 10 – 13.

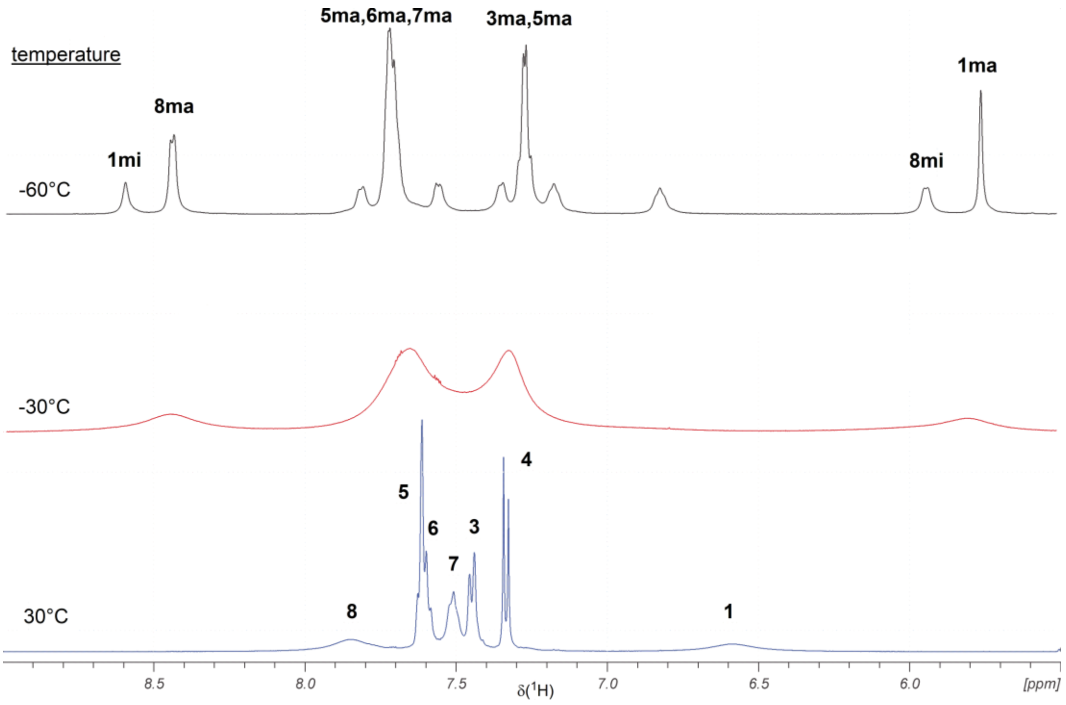

Figure S9. ^{1}H NMR spectrum of ***rac*-DFSN-Br$_2$ 5** in CD_2Cl_2 at different temperatures. The ratio of major (ma) and minor (mi) rotamer is about 70 : 30 at -60°C.

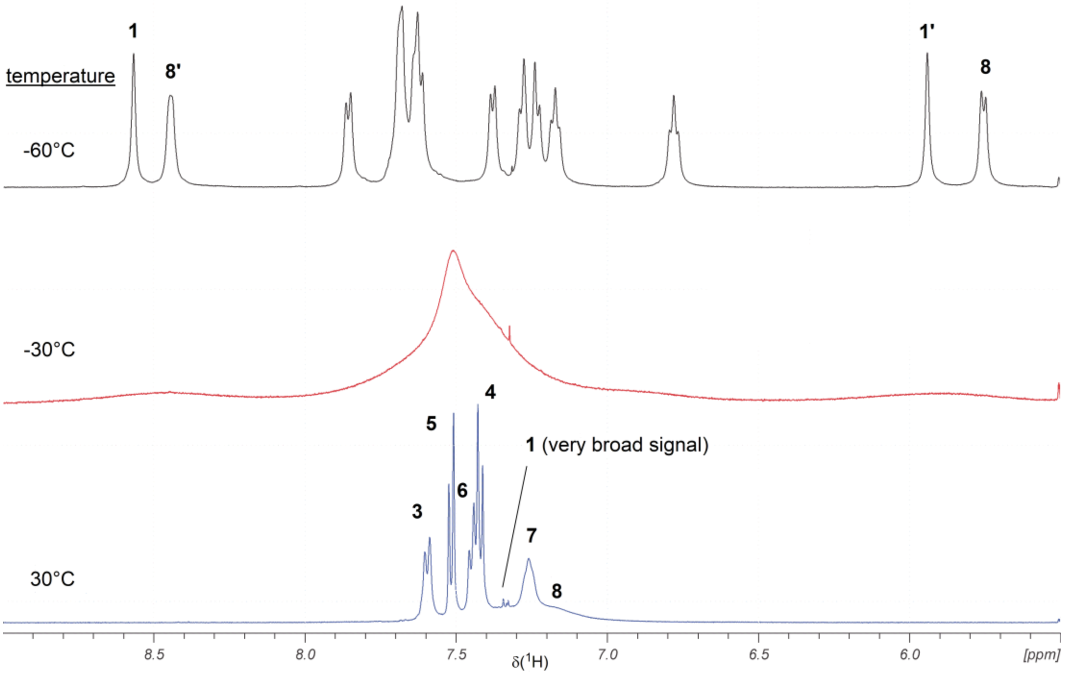

Figure S10. ^{1}H NMR spectrum of ***meso*-DFSN-Br$_2$ 5** in CD_2Cl_2 at different temperatures.

3.3 NMR spectra of P*mmp*P-DFSN$_x$ polymers

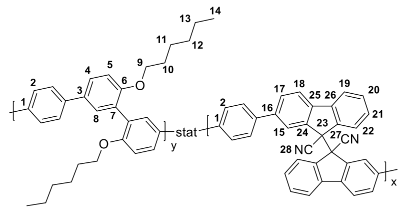

P*mmp*P-DFSN$_x$

P$_x$, x = 0, 0.25, 1, 2, 3, 4, 20 (molar content of DFSN)

x + y = 100 mol%

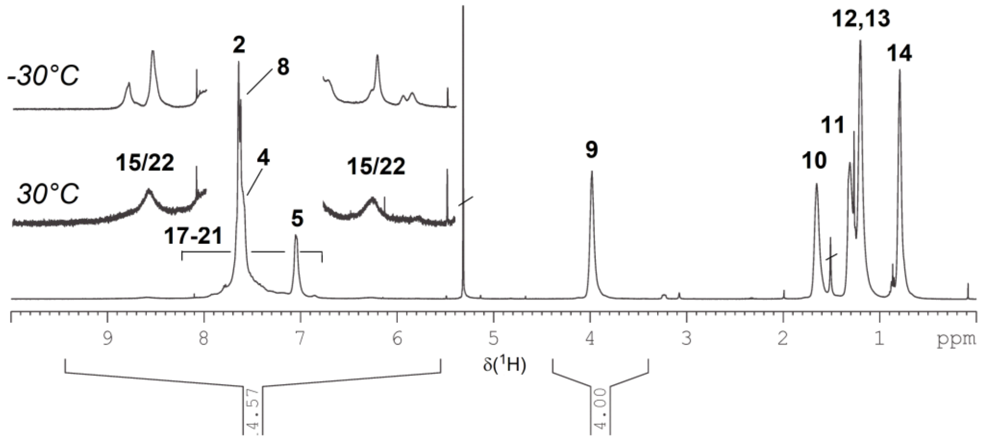

Figure S11. ^{1}H NMR spectrum of P*mmp*P-DFSN$_{20}$ (**P$_{20}$**) in CD_2Cl_2 at 30°C. The insets show the signals of H_{15} and H_{22} with different chemical shifts depending on the stereochemistry. These signals become narrower with decreasing temperature, as shown for -30°C.

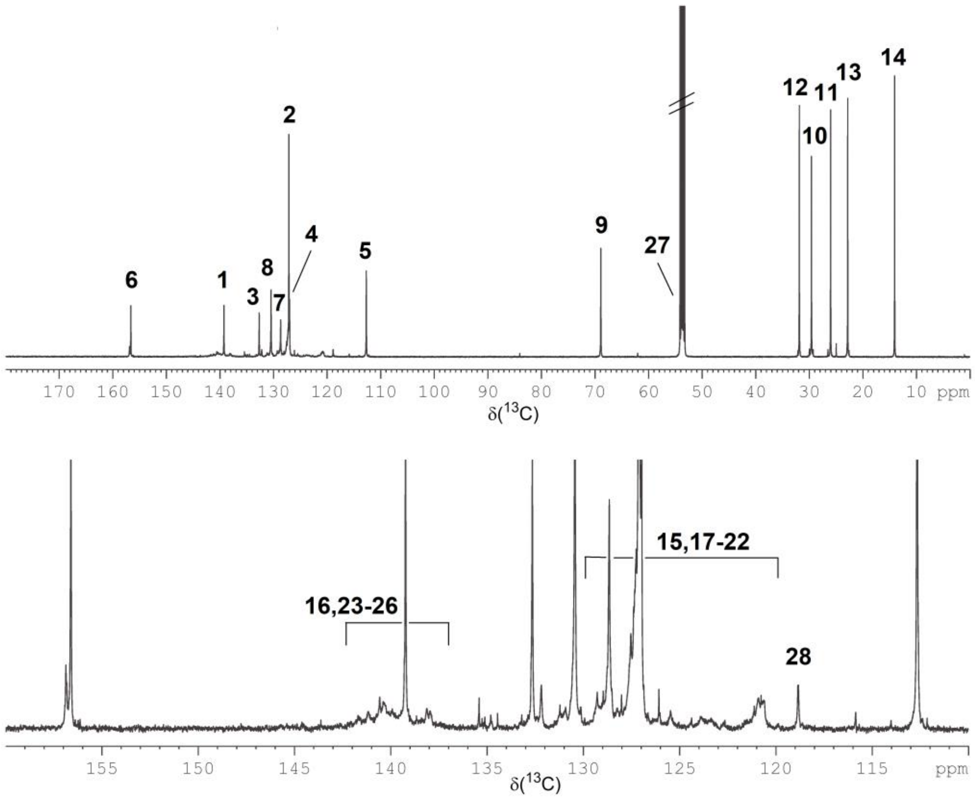

Figure S12. ^{13}C NMR spectrum of P*mmp*P-DFSN$_{20}$ (**P$_{20}$**) in CD_2Cl_2 at 30°C.

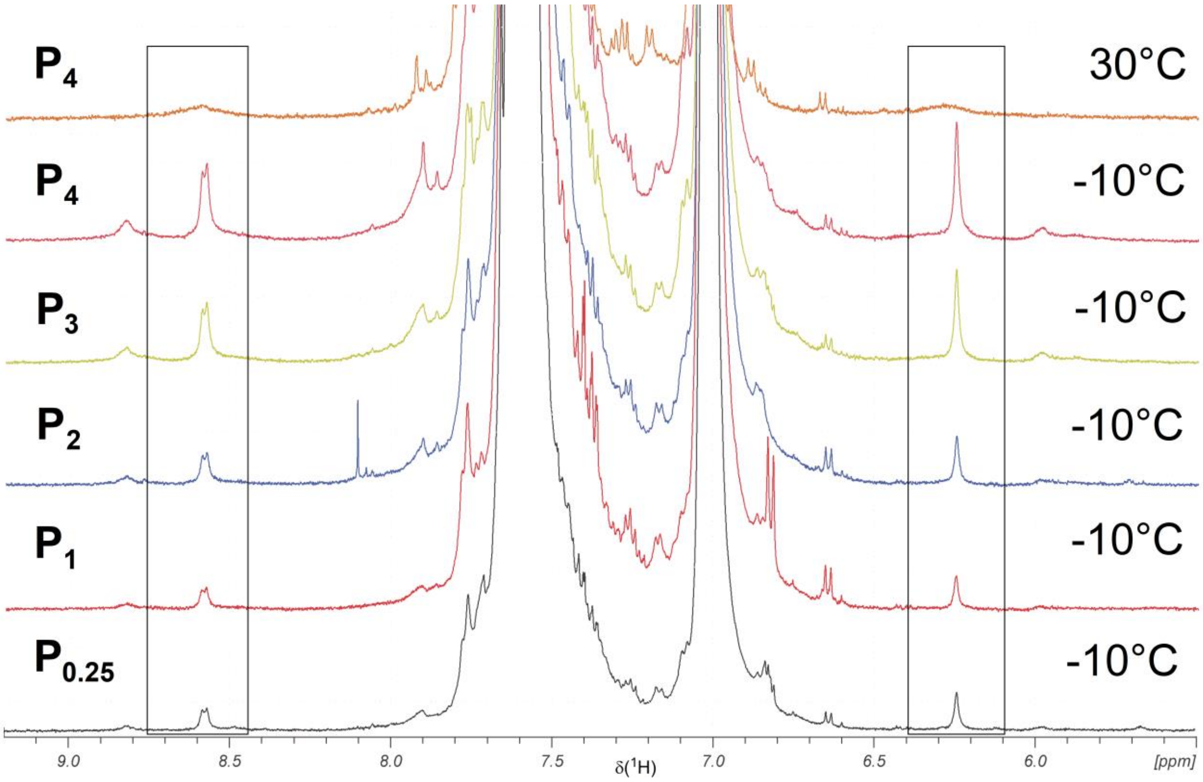

Figure S13. ^{1}H NMR spectra of **P*mmp*P- DFSN$_x$** polymers **P$_{0.25}$**, **P$_1$**, **P$_2$**, **P$_3$** and **P$_4$** in CD_2Cl_2 at -10°C. Additionally, the spectrum of **P$_4$** at 30°C is shown. The spectra are normalised to the same signal intensity of the OCH_2 signal (Note: normalization should be performed on the Ph-Bpin$_2$-based signal H_2, as only this component is constant for all polymers. However, this signal is superimposed (see **Figure S11**). The selected OCH_2 signal changes between 99.75 (**P$_{0.25}$**) and 96 (**P$_4$**) and should allow a qualitative comparison of the spectra).

The framed signal areas result from **DFSN** comonomer (signal assignment to diastereomer unknown). The gradual increase from **P$_1$** to **P$_4$** is obvious. This does not apply to **P$_{0.25}$**, where a larger than expected signal intensity was observed.

4. SEC curves

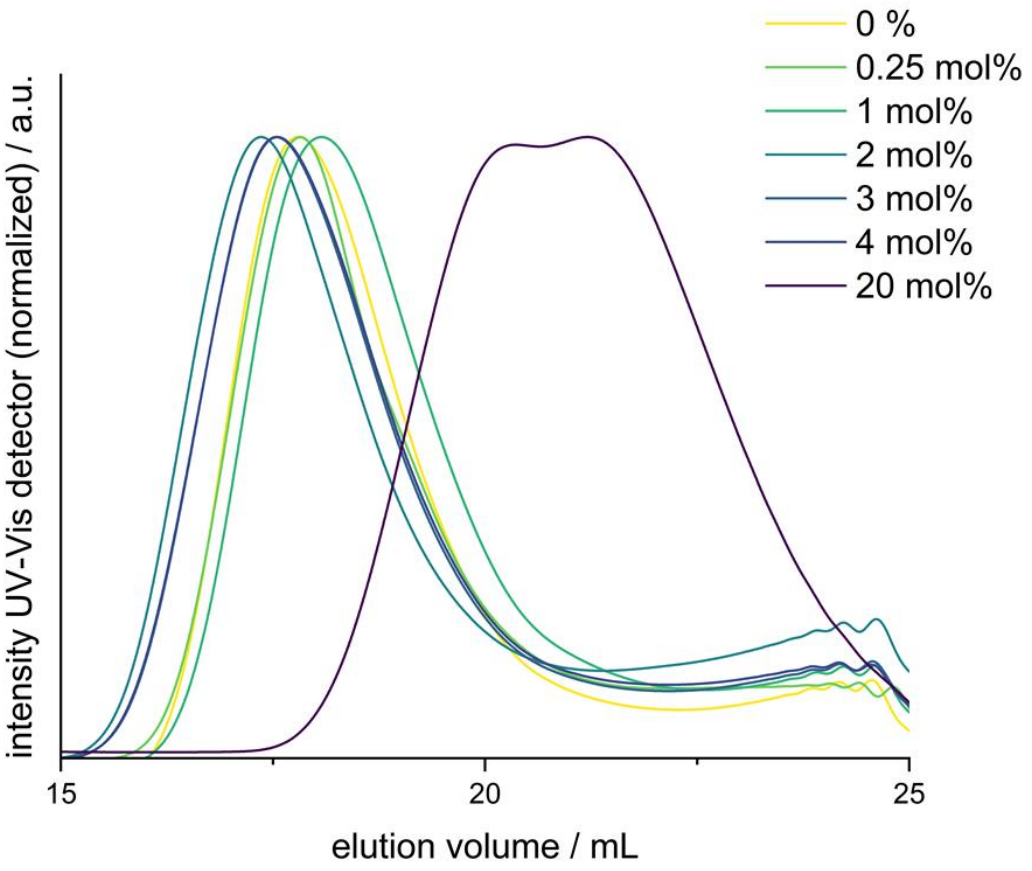

Figure S14. Normalized SEC elution curves of samples of P*mmp*P-DFSN$_x$ (**P$_x$**) with covalently incorporated DFSN-Br$_2$ **5** showing distributions with molecular weights exceeding 90 kg·mol^{-1} (M_w) for samples **P$_0$** to **P$_4$**.

5. Thermal characterization

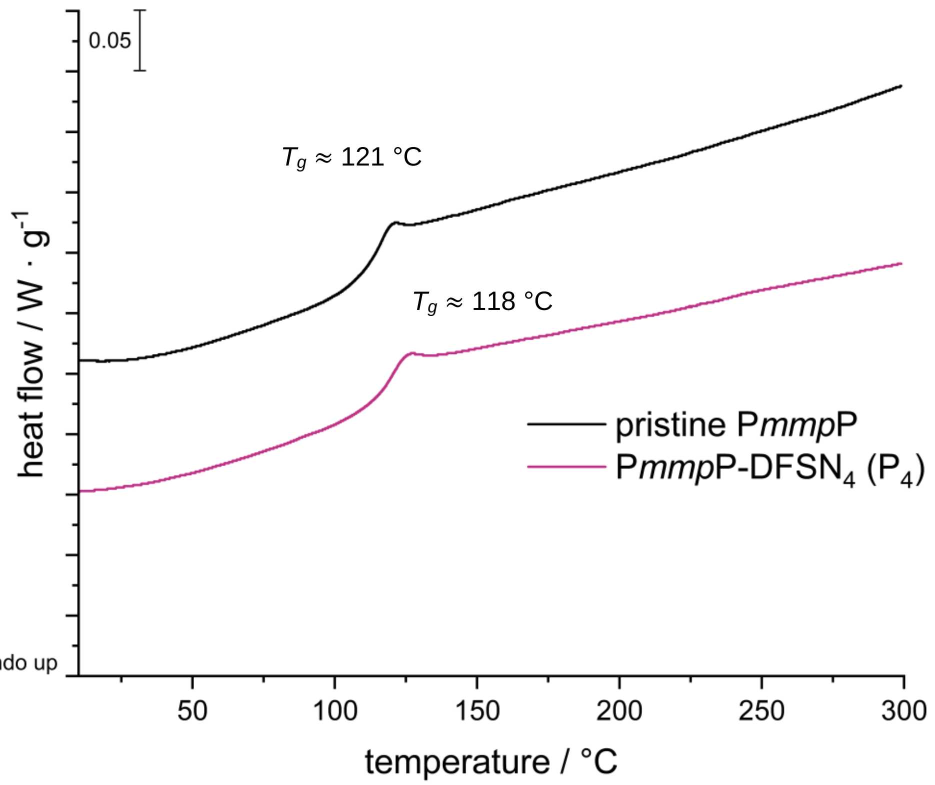

Figure S15. Differential Scanning Calorimetry (DSC) 2. heating scan of **P_4** (heating/cooling rate 10 °C·min^{-1}) and 2. heating scan of pristine P*mmp*P (heating/cooling rate 10 °C·min^{-1}). T_g for pristine P*mmp*P was first reported by KEMPE *et al.*[1]

6. Mechano-optical characterization

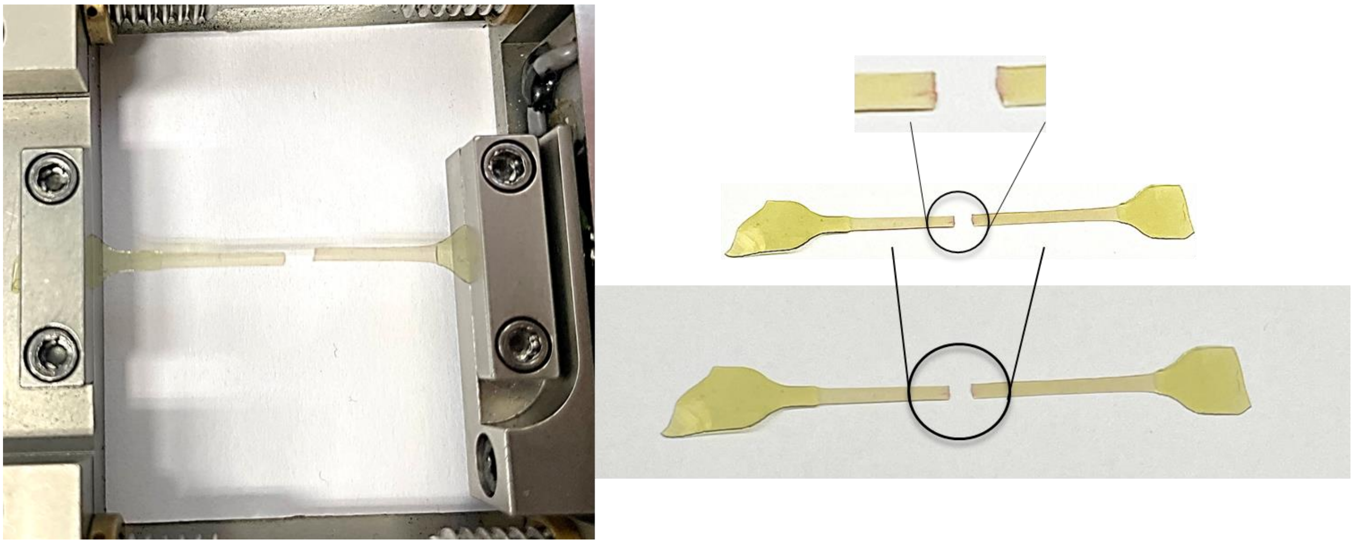

Figure S16. Stress-strain setup for the bone shaped polymer sample displaying the enlarged break area of the sample on the right.

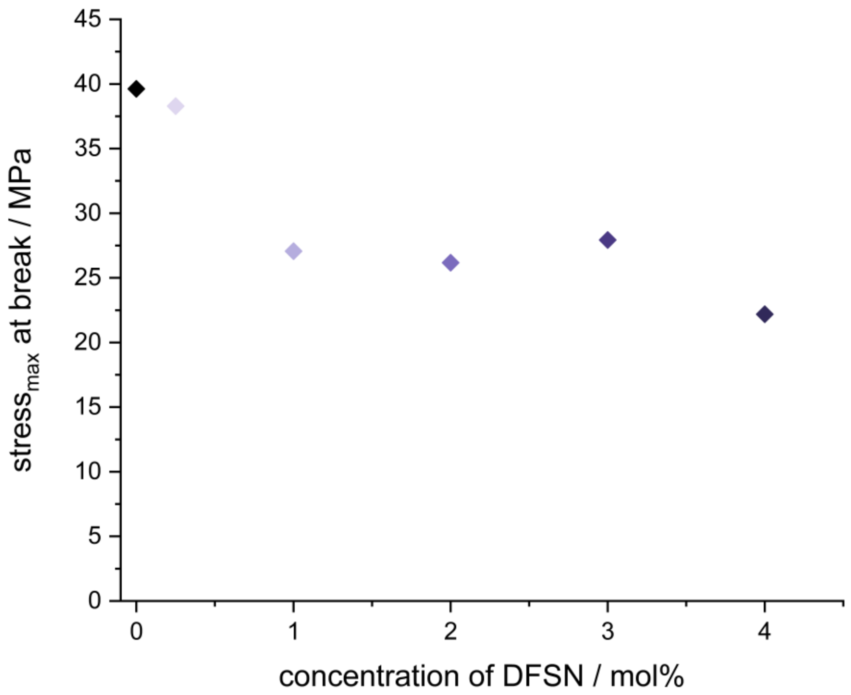

Figure S17. Maximum stress versus DFSN concentration.

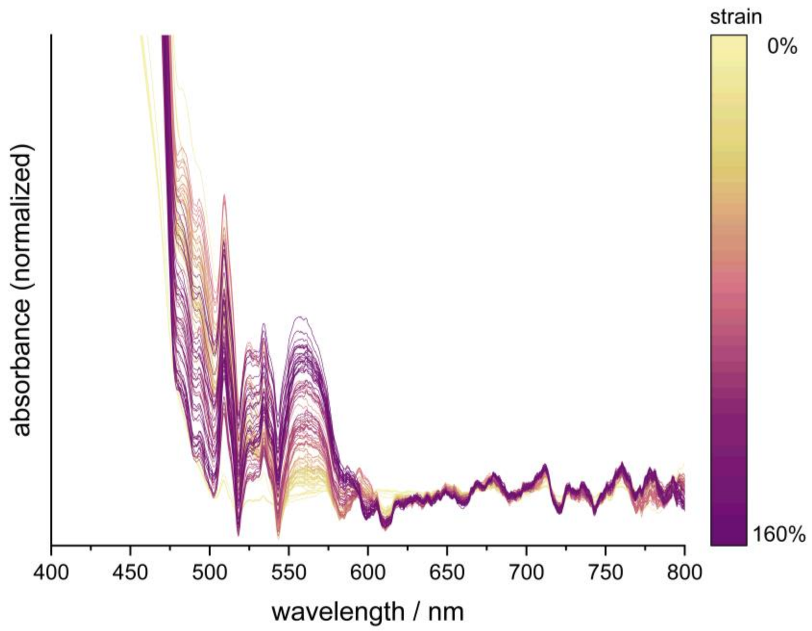

Figure S18. Normalized UV-vis absorbance of P*mmp*P-DFSN$_2$ (**P$_2$**) during tensile testing. The absorbance data was baseline corrected, smoothed with Savitsky-Golay smoothing using third order polynomial and normalized.

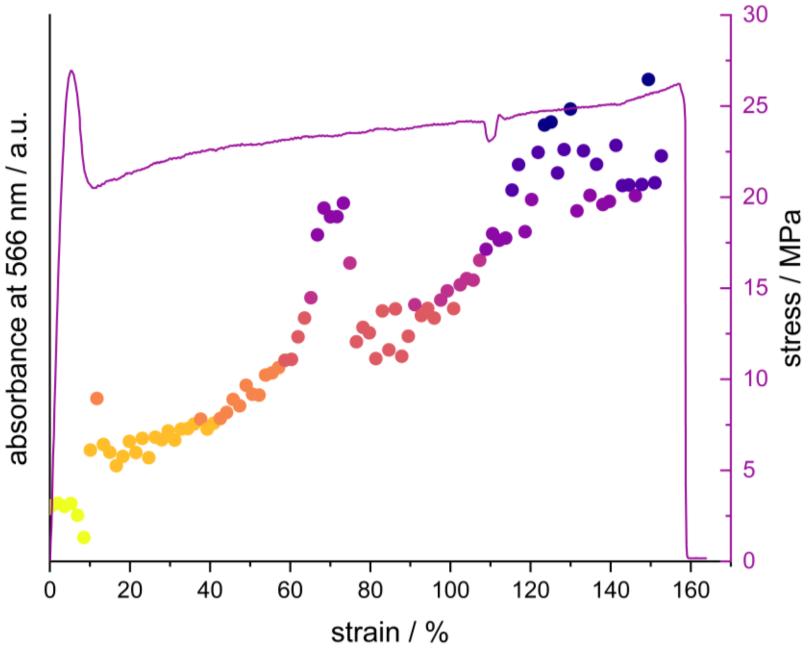

Figure S19. In-situ absorbance at 556 nm as a function of strain of PmmpP-DFSN$_2$ (**P$_2$**) showing that DFSN is a suitable force probe and that stress transduction occurs after sample necking.

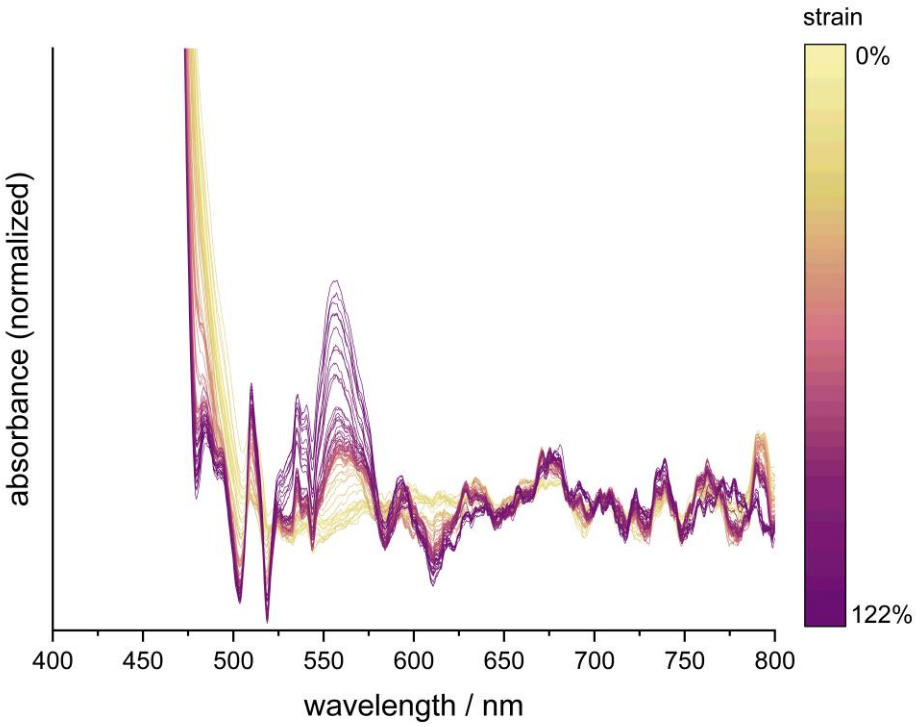

Figure S20. Normalized UV-vis absorbance of P*mmp*P-DFSN$_3$ (**P$_3$**) during tensile testing. The absorbance data was baseline corrected, smoothed with Savitsky-Golay smoothing using third order polynomial and normalized.

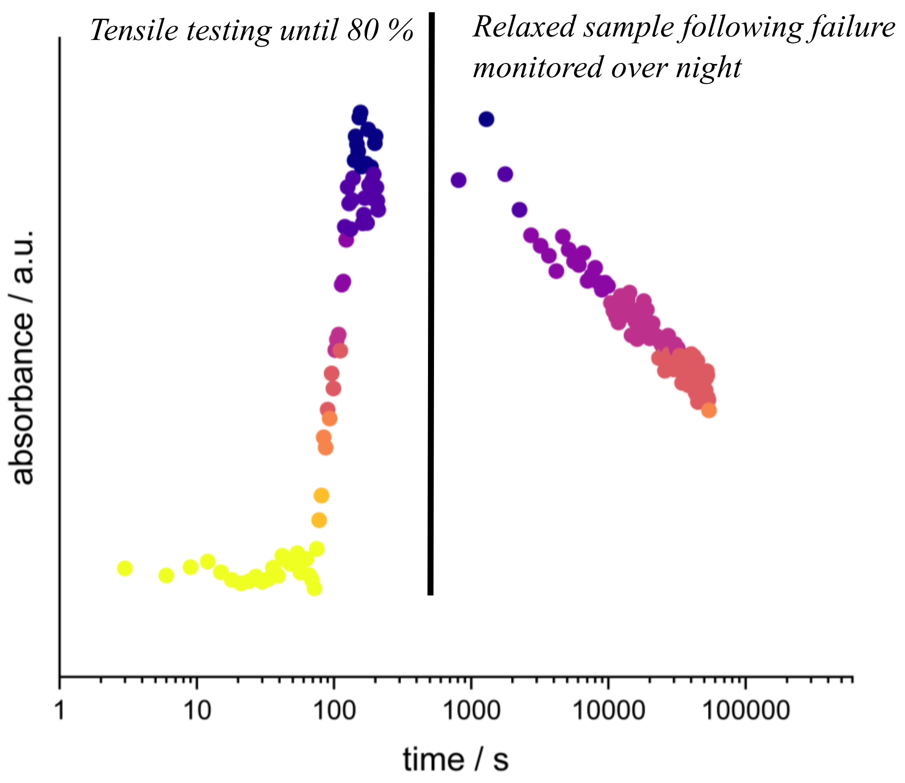

Figure S21. In-situ absorbance at 556 nm during a stress-strain experiment of P*mmp*P-DFSN$_3$ (P$_3$) until 80% strain. The specimen was kept in the setup. The specimen failed 60 s later allowing for relaxation, and a sample area near the failed region was monitored overnight via in-situ UV-vis spectroscopy.

7. EPR data and reversibility

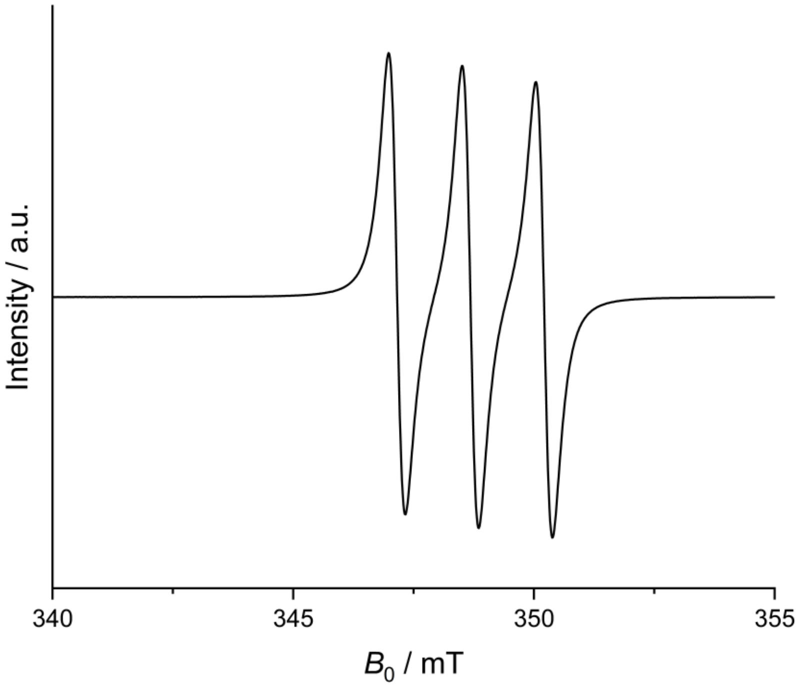

Figure S22. Electron paramagnetic resonance (EPR) spectrum of TEMPOL radical, g = 2.0057[3], which was used as calibration standard for the magnetic field, taking a new measurement for each measuring day. The microwave power was 0.6 mW, the modulation frequency 100 kHz and the modulation amplitude 0.1 mT. The microwave frequency was corrected to 9.81 GHz.

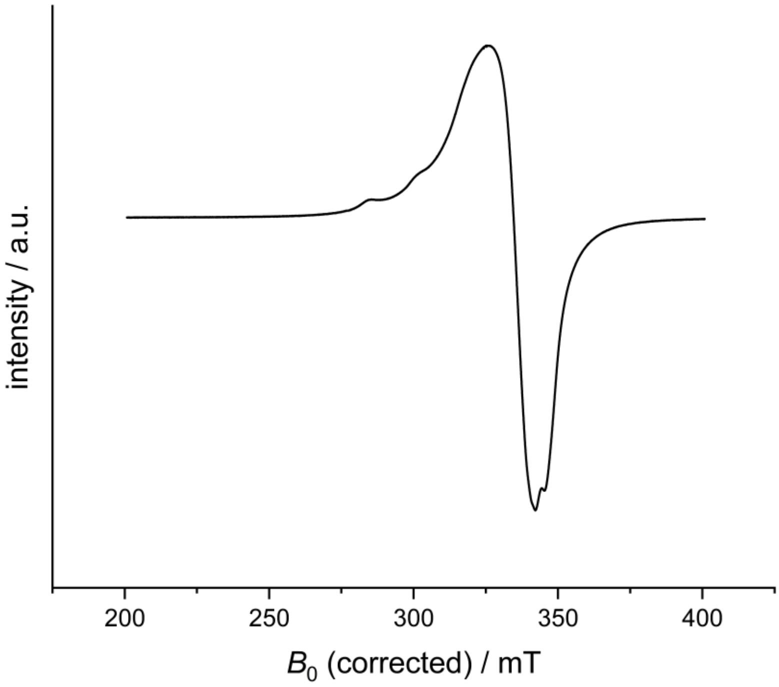

Figure S23. Electron paramagnetic resonance (EPR) spectra of $Cu(acac)_2$ ground with SiO_2 used for intensity calibration of the measured P*mmp*P-DFSN$_{20}$ (**P$_{20}$**). For each measuring day and spectrum of P_{20} one spectrum of $Cu(acac)_2$ under the same conditions was recorded, the doubly integrated intensity of P_{20} was then divided by the doubly integrated intensity of $Cu(acac)_2$. The microwave power was 1.6 mW, the modulation frequency 100 kHz and the modulation amplitude 0.1 mT. The microwave frequency was corrected to 9.81 GHz.

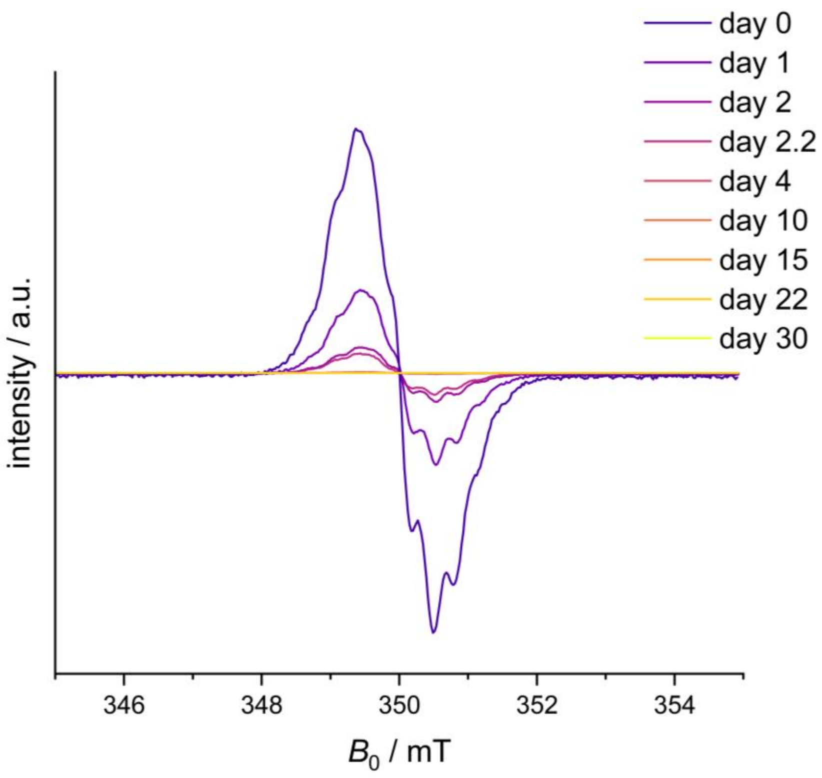

Figure S24. Decrease of intensity of ground P*mmp*P-DFSN20 (**P20**) over a time period of 30 days, magnetic field corrected with TEMPOL and intensity calibrated with $Cu(acac)_2$. The remains of the characteristic fingerprint curve of **P20**, even after the relative amount in **Figure 3** b) dropped under 1% are assigned to the high sensitivity of the measurement method. The microwave power was 1.6 mW, the modulation frequency 100 kHz and the modulation amplitude 0.1 mT. The microwave frequency was corrected to 9.81 GHz. The number of scans was increased with continued measurements for better signal to noise ratio, and the intensity therefore divided by the number of scans before plotting.

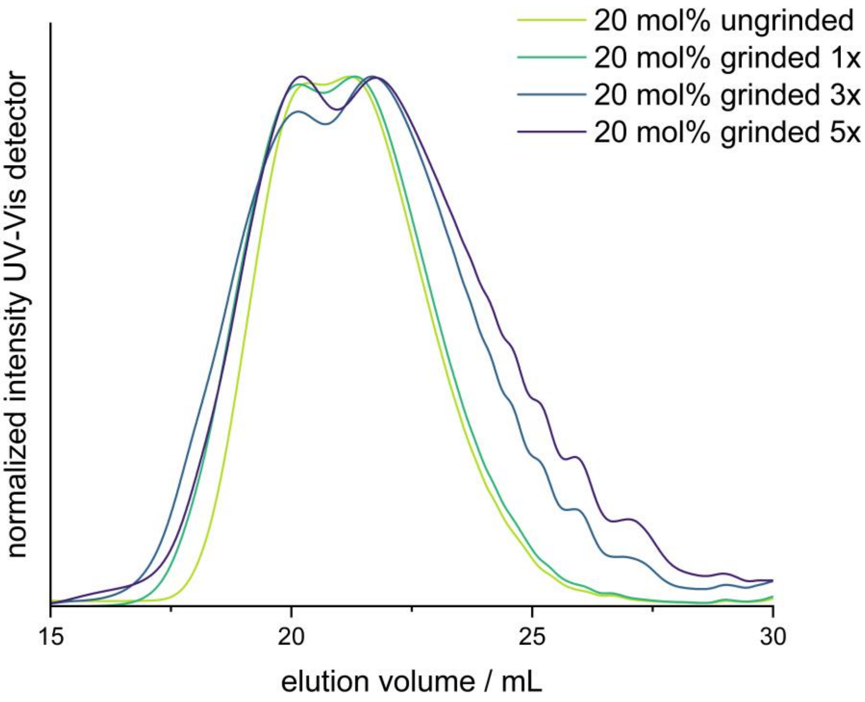

Figure S25. SEC elution curves of P*mmp*P-DFSN$_{20}$ (**P$_{20}$**) which was ground five times via ball milling experiment and resolved in THF.

P*mmp*P-DFSN$_{20}$ (**P$_{20}$**) was ground five times via ball milling. For the process, five balls with 0.5cm diameter each were used, one grinding process took 10 minutes. After each grinding process, SEC in THF was measured. The comparison of the data shows a broadening of the SEC elution curves, but no decline, thus supporting the assumption of the recombination of opened polymer chain radical ends. Simple solution experiments support this thesis, as freshly ground, pink colored **P$_{20}$** turns yellow immediately in solution upon being dissolved in tetrahydrofurane, dichloromethane or chloroform.

8. Supporting references

(1) Kempe, F.; Brügner, O.; Buchheit, H.; Momm, S. N.; Riehle, F.; Hameury, S.; Walter, M.; Sommer, M. A Simply Synthesized, Tough Polyarylene with Transient Mechanochromic Response. *Angew. Chem. Int. Ed.* **2018**, *57* (4), 997–1000. https://doi.org/10.1002/anie.201709142.

(2) Horatz, K.; Giampà, M.; Karpov, Y.; Sahre, K.; Bednarz, H.; Kiriy, A.; Voit, B.; Niehaus, K.; Hadjichristidis, N.; Michels, D. L.; Lissel, F. Conjugated Polymers as a New Class of Dual-Mode Matrices for MALDI Mass Spectrometry and Imaging. *J. Am. Chem. Soc.* **2018**, *140* (36), 11416–11423. https://doi.org/10.1021/jacs.8b06637.

(3) Marshall, D.; Christian, M.; Gryn'ova, G.; Coote, M.; Barker, P.; Blanksby, S. Oxidation of 4-Substituted TEMPO Derivatives Reveals Modifications at the 1-and 4-Positions. *Org. Biomol. Chem.* **2011**, *9*, 4936–4947. https://doi.org/10.1039/c1ob05037k.

(4) Raisch, M.; Maftuhin, W.; Walter, M.; Sommer, M. A Mechanochromic Donor-Acceptor Torsional Spring. *Nat. Commun.* **2021**, *12* (1), 4243. https://doi.org/10.1038/s41467-021-24501-1.

(5) Dougherty, D. A.; Llort, F. M.; Mislow, K.; Blount, J. F. Conformational Analysis of Tetraarylethanes. *Tetrahedron* **1978**, *34* (9), 1285–1300. https://doi.org/10.1016/0040-4020(78)88322-7.

(6) Olah, G. A.; Field, L. D.; Watkins, M.; Malhotra, R. Crowded Hydrocarbons. 2. Conformational Study of 9,9'-Bifluorenyls by Dynamic Nuclear Magnetic Resonance Spectroscopy. *J. Org. Chem.* **1981**, *46* (9), 1761–1764. https://doi.org/10.1021/jo00322a001.
